# Vanillin derived a carbonate dialdehyde and a carbonate diol: novel platform monomers for sustainable polymers synthesis†

**DOI:** 10.1039/c8ra07185c

**Published:** 2018-10-05

**Authors:** De Bai, Qin Chen, Yang Chai, Tianhua Ren, Caijuan Huang, Ian D. V. Ingram, Michael North, Qiang Zheng, Haibo Xie

**Affiliations:** Department of Polymeric Materials & Engineering, College of Materials & Metallurgy, Guizhou University West Campus, Huaxi District Guiyang P. R. China 550025 hbxie@gzu.edu.cn; Green Chemistry Centre of Excellence, Department of Chemistry, University of York York UK YO10 5DD

## Abstract

Vanillin has been regarded as one of the important biomass-based platform chemicals for aromatic polymers synthesis. Herein, novel symmetric bis(4-formyl-2-methoxyphenyl)carbonate (BFMC) and bis(4-(hydroxymethyl)-2-methoxyphenyl)carbonate (BHMC) polymeric monomers have been synthesized in high yields using vanillin as a raw chemical, which have been submitted for polymer synthesis *via* well-established polymeric strategies. A new class of poly(carbonate ester)s oligomers with amide moieties in their side chain can be prepared by using the BFMC as one of monomers *via* the Passerini three compound reaction (3CR). A new class of poly(carbonate ester)s oligomers and poly(carbonate urethane)s can be prepared *via* reactions between BHMC with dicarboxylic acid chlorides and diisocyanates, respectively. Their structure have been confirmed by ^1^H NMR, ^13^C NMR and FTIR, and the gel permeation chromatograph (GPC) analysis shows that the Mn of poly(carbonate ester)s oligomers ranges from 3100 to 7900 with PDI between 1.31 and 1.65, and the Mn of poly(carbonate urethane)s ranges from 16 400 to 24 400 with PDI ranging from 1.36 to 2.17. The DSC analysis shows that the poly(carbonate ester)s oligomers have relative low *T*_g_ ranging from 37.4 to 74.1 °C, and the poly(carbonate urethane)s have *T*_g_ ranging from 97.3 to 138.3 °C, mainly correlating to the structure of dicarboxylic acid chlorides and diisocyanates used.

## Abstract 2

Novel classes of lignin-derived poly(carbonate ester)s, poly(carbonate ester)s pending amide moiety oligomers, and poly(carbonate urethane)s have been designed and synthesized.

## Graphical abstract

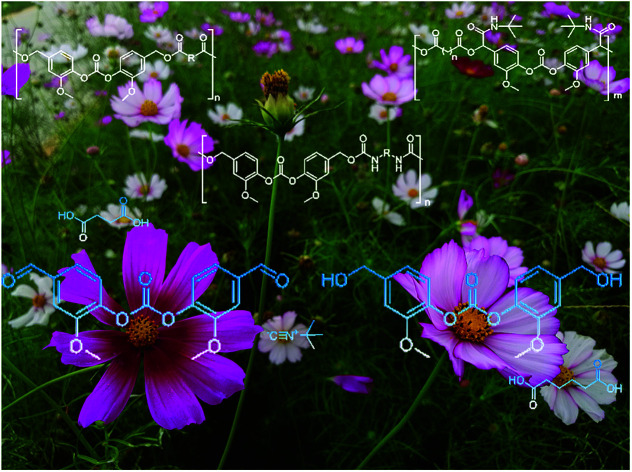

## Introduction

The development of modern society relies heavily on polymeric materials, furthermore, most of these polymers are currently prepared from petrochemical resources. However, the depletion of fossil resources and the increasing environmental concerns have motived researches to prepare sustainable and environmental friendly polymers by using renewable resources, such as lignocellulosic biomass and CO_2_ as feedstocks.^1,2^ The pursuit of design and preparation of polymers from lignocellulosic biomass and CO_2_ mainly owes to their carbon neutral characteristics and great abundance, thus being able to prepare polymers with low carbon footprint compared to traditional synthetic polymers;^3^ at the same time, the particular chemical diversity of biomass-based monomers and the specific molecular structure of CO_2_ determine the as-prepared polymers presenting different and particular chemical and physical properties.^4^ The bloom of this area was significantly promoted by the rapid development of modern catalytic biorefineries, which has produced a big library of chemicals without or with further upgrading ready for polymer synthesis.^5^ Furthermore, the development, properties design and application potential of bio-based synthetic polymers will greatly depend on the rational monomer design by taking the advantages of bio-based chemicals' inherent structures of and controllable polymerization methods.^6^

Lignin is one of the main components in lignocellulosic biomass,^7^ being as the most abundant renewable sustainable aromatic chemical source, its chemical catalytic conversion produced a spectrum of aromatic chemicals,^8^ among of which vanillin has been produced commercially.^9,10^ Recently, vanillin and its upgrading chemicals (*e.g.* creosol) have been regarded as one of important platform molecular for the design and synthesis new bio-based synthetic polymers.^11^ For example, a preliminary study of the Perkin reaction and hydrogenation of vanillin has afforded acetyl dihydroferulic acid and the following polymerization resulted in poly(dihydroferulic acid), which exhibits similar thermal property to PET.^12^ Vanillin based monomers containing epoxy, cyclic carbonates, allyl, amine, alcohol and carboxylic acid groups were prepared and could be used for epoxy, polyester, polyurethanes and non-isocyanate polyurethanes polymers. Cramail *et al.* synthesized vanillin-based diepoxides and the following curing afford vanillin-based epoxides with excellent flame retardancy and high *T*_g_ together with outstanding mechanical properties.^13^

Polycarbonates are known for their excellent thermal stability and mechanical properties, and have been the most useful engineering plastics,^14,15^ which is one of important strategies to use CO_2_ as renewable feedstock. Commonly, the polycarbonate was prepared by interfacial polycondensation of bisphenol A and phosgene or ring-opening polymerization of epoxides and CO_2_. Their performance can be further improved or tuned by the synthesis of mixed copolymers, such as poly(carbonate-ester) as well as poly(carbonate-urethane).^16,17^ because of the co-existing of carbonate, ester or urethane functional moiety in one polymer chain.^18^ Particular, the studies of bio-based polycarbonate have been obtained much attentions, and have significantly extended the family of polycarbonates with versatile properties.^4,19–22^

Herein, two polymeric monomers, bis(4-formyl-2-methoxyphenyl)carbonate (BFMC) and bis(4-(hydroxymethyl)-2-methoxyphenyl)carbonate (BHMC) were designed and prepared in high yield by taking the advantages of phenolic hydroxyl and aldehyde group in vanillin, and the polymerization potential of them have been investigated through traditional well-established methods. Furthermore, their structures have been elucidated by various characteristic technologies, and the thermo-properties also have been evaluated by DSC and TGA.

## Experimental section

### Materials

All of the synthesis and manipulations of air- and moisture-sensitive materials were carried out in flask or Schlenk-type bottles in a high-vacuum environment or under nitrogen protection. *tert*-Butyl isonitrile were purchased from Energy Chemical and used as received. Vanillin, succinic acid, hexanedioic acid, succinyl chloride (SC), terephthaloyl chloride (TC), isophorone diisocyanate (IPDI), diphenylmethane diisocyanate (MDI), pyridine and 1,8-diazabicyclo[5.4.0]undec-7-ene (DBU) were purchased from Aladdin and used as received. Triphosgene was purchased from Macklin. Sodium borohydride (NaBH4) was purchased from Kemel. Other common organic solvents are commercially available. Dichloromethane, tetrahydrofuran and triethylamine are distilled with calcium hydride to remove water before use and then stored in a vacuum drying oven over activated Davison 4 Å molecular sieves, while other chemicals were used as received.

### Instrumentations

NMR spectra were recorded on a JOEL ECX500 spectrometer (400 MHz for ^1^H NMR and 101 MHz for ^13^C NMR). Chemical shifts for ^1^H and ^13^C NMR spectra were referenced in ppm relative to tetramethylsilane with the solvent residual resonances as the internal standard (DMSO-d_6_, *δ* 2.50 ppm for ^1^H NMR and 39.52 ppm for ^13^C NMR; CDCl_3_, *δ* 7.26 ppm for ^1^H NMR and 77.16 ppm for ^13^C NMR). Fourier transform infrared (FTIR) spectroscopy was performed on a Thermoscientific (Nicolet iS50) FTIR spectrometer at room temperature in the range of 500–4000 cm^−1^. The number-average molecular weight (*M*_n_) and dispersity (*Đ* = *M*_w_/*M*_n_) were measured by gel permeation chromatography (GPC) with THF or DMF as the eluent. The instrument was calibrated with 10 PMMA standards, and chromatograms were processed with Waters Empower software. Thermal properties of the polymers were measured by thermal gravimetric analyzer (TGA) and differential scanning calorimetry (DSC) analyzer under dry nitrogen flow of 40 mL min^−1^. For TGA analysis, polymer samples were heated from ambient temperature to 750 °C at a heating rate of 10 °C min^−1^ under anitrogen atmosphere. For DSC analysis, polymer samples were first heated from room temperature to 180 °C at 10 °C min^−1^, equilibrated at this temperature for 5 min, then cooled down to −90 °C at 10 °C min^−1^, held at this temperature for 5 min, and reheated to 250 °C at 10 °C min^−1^. The glass transition temperature (*T*_g_) were obtained from the second heating cycle, after removing the thermal history of the samples.

### General procedure for synthesis of BFMC (1a)

To a solution of vanillin (0.913 g, 6 mmol) in CH_2_Cl_2_ (20 mL) was added triethylamine (1.25 mL, 9 mmol), and the mixture was stirred at 0 °C for 10 min. A solution of triphosgene (98%, 0.303 g, 1 mmol) in CH_2_Cl_2_ (5 mL) was added to the mixture, and the resulting mixture was stirred at 0 °C for 2 h. The reaction was quenched with 1 M HCl and then washed to neutral with distilled water. The inorganic phase is extracted with dichloromethane (3 × 40 mL), combined organic phase and dried over anhydrous Na_2_SO_4_. The residue was purified by recrystallized from dichloromethane/hexane to give compound a as a white solid. Yield: 93%. Mp = 95–96 °C, ^1^H NMR (400 MHz, CDCl_3_), *δ* (ppm) = 9.97 (s, 2H, –CHO), 7.54–7.42 (m, 6H, aromatic protons), 3.98 (s, 6H, –OCH_3_). ^13^C NMR (125 MHz, CDCl_3_) *δ* (ppm) = 190.99, 151.95, 149.93, 144.66, 135.73, 124.69, 122.93, 111.32, 56.41.

### General procedure for synthesis of BHMC (1c)

In a 25 mL flask, BFMC (1.651 g, 5 mmol) was dissolved in dehydrated tetrahydrofuran (15 mL) and kept at 0 °C in an ice bath. Sodium borohydride (0.567 g, 15 mmol) was added to the flask slowly over 10 min, then the reaction mixture was quenched with dilute hydrochloric acid. The solution was extracted with dichloromethane (3 × 20 mL) and washed with brine, then separated and dried over anhydrous Na_2_SO_4_. The residue was purified by recrystallized from ethyl acetate/petroleum ether to give a white solid. Yield: 86%. Mp = 125–127 °C, ^1^H NMR (400 MHz, DMSO-d_6_) *δ* (ppm) = 7.21–6.92 (m, 6H, aromatic protons), 5.26 (t, 2H, –OH), 4.50 (d, 4H, –CH_2_), 3.84 (s, 6H, –OCH_3_). ^13^C NMR (101 MHz, DMSO-d_6_) *δ* (ppm) = 150.97, 150.45, 142.31, 138.16, 121.68, 118.26, 111.18, 62.52, 55.89.

### General procedure for synthesis of PCEAs

BFMC (0.330 g, 1 mmol), diacids (1 mmol) and *tert*-butyl isonitrile (230 μL, 2 mmol) were added sequentially to a 25 mL Schlenk flask, then added suitable solvent to dissolve the mixture. The mixed system reacted at 40 °C under nitrogen atmosphere. After the reaction was completed, the mixture was poured into a large amount of ethyl ether to regenerate the polymer and washed three times with benzene, collected by centrifugation, then dried at 50 °C under vacuum for 24 h.

#### PCEA-1

Succinic acid (0.119 g, 1.0 mmol), isolated yield (77.3%). ^1^H NMR (400 MHz, DMSO-d_6_) *δ* (ppm) = 7.84 (s, 2H, –NH–). 7.31–7.08 (m, 6H, aromatic protons), 5.83 (s, 2H,–CH–), 3.85 (s, 6H, –OCH_3_), 2.72 (s, 4H, –CH_2_–), 1.21 (s, 18H, –CH_3_). ^13^C NMR (101 MHz, DMSO-d_6_) *δ* (ppm) = 171.16, 166.69, 150.45, 150.14, 139.26, 135.82, 122.19, 119.46, 112.05, 74.75, 56.06, 50.56, 28.58, 28.35.

#### PCEA-2

Hexanedioic acid (0.146 g, 1.0 mmol), isolated yield (94.8%). ^1^H NMR (400 MHz, DMSO-d_6_) *δ* (ppm) = 7.88 (s, 2H, –NH–), 7.31–7.08 (m, 6H, aromatic protons), 5.82 (s, 2H, –CH–), 3.85 (s, 6H, –OCH_3_), 2.45 (s, 4H, –CH_2_–), 1.60 (s, 4H, –CH_2_–), 1.21 (s, 18H, –CH_3_). ^13^C NMR (101 MHz, DMSO-d_6_) *δ* (ppm) = 172.03, 166.89, 150.68, 150.45, 139.27, 135.91, 128.39, 122.21, 119.57, 112.12, 74.47, 56.07, 50.51, 33.00, 28.36, 23.79.

### General procedure for synthesis of PCEs

To a stirred THF solution of compound BHMC (0.334 g, 1.0 mmol) and diacyl chloride (1.0 mmol) was added dropwise a THF solution of pyridine (0.177 g, 2.2 mmol) at room temperature under nitrogen atmosphere in a 25 mL two-necked flask. After 24 h, the reaction mixture was poured into a large amount of methanol to regenerate the polymers, and the resulting PEs were collected by centrifugation and dried at 50 °C under vacuum for 24 h.

#### PCE-1

Succinyl chloride (116 μL, 1.0 mmol, 95%), isolated yield: 82.4%. ^1^H NMR (400 MHz, CDCl_3_) *δ* (ppm) = 7.20–6.91 (m, 6H, aromatic protons), 5.08 (s, 4H, –OCH_2_–), 3.88 (s, 6H, –OCH_3_), 2.70 (s, 4H, –CH_2_–). ^13^C NMR (101 MHz, DMSO-d_6_) *δ* (ppm) = 171.91, 150.71, 150.60, 139.03, 135.86, 122.16, 120.01, 112.75, 65.17, 56.02, 28.64.

#### PCE-2

Terephthaloyl chloride (0.203 g, 1.0 mmol), isolated yield (71.9%). ^1^H NMR (400 MHz, DMSO-d_6_) *δ* (ppm) = 8.09 (s, 4H, aromatic protons), 7.33–7.09 (m, 6H, aromatic protons), 5.34 (s, 4H, –OCH_2_–), 3.84 (s, 6H, –OCH_3_). ^13^C NMR (101 MHz, DMSO-d_6_) *δ* (ppm) = 165.38, 151.20, 150.93, 139.77, 136.01, 134.06, 130.13, 122.82, 120.95, 113.73, 66.86, 56.63.

### General procedure for synthesis of PCUs

BHMC (0.334 g, 1 mmol) and diisocyanate (1 mmol) were dissolved in 5 mL of THF in a 25 mL two-necked flask, to which DBU (3 mol%) was added. The reaction mixture was placed in the oil bath reaction 24 h. The reaction mixture was poured into a large amount of ethyl ether to regenerate the polymers and washed three times with ethanol, collected by centrifugation, then dried at 50 °C under vacuum for 24 h.

#### PCU-1

MDI (210 μL, 1.0 mmol), isolated yield (73%). ^1^H NMR (400 MHz, DMSO-d_6_) *δ* (ppm) = 9.72 (s, 2H, –NH–), 7.38–7.04 (m, 14H, aromatic protons), 5.13 (s, 4H, –ArCH_2_O–), 3.86 (s, 6H, –OCH_3_), 3.79 (s, 2H, –ArCH_2_Ar–). ^13^C NMR (400 MHz, DMSO-d_6_) *δ* (ppm) = 153.35, 150.81, 150.67, 139.13, 136.42, 135.68, 129.11, 128.97, 122.26, 120.34, 118.36, 113.08, 65.36, 56.12, 41.30.

#### PCU-2

IPDI (210 μL, 1.0 mmol), isolated yield (67%).^1^H NMR (400 MHz, DMSO-d_6_) *δ* (ppm) = 7.34 (s, H, –NH–), 7.27–6.96 (m, 6H, aromatic protons), 6.71 (s, H, –NH–), 5.02 (s, 4H, –ArCH_2_O–), 3.84 (s, 6H, –OCH_3_), 3.60 (br, 1H, NH–CH), 2.76 (m, 2H, NH–CH_2_), 1.76 (s, 2H, –CH_2_–), 1.48 (s, 2H, –CH_2_–), 1.09–0.81 (m, 11H, –CH_2_– and –CH_3_). ^13^C NMR (101 MHz, DMSO-d_6_) *δ* (ppm) = 156.70, 155.23, 150.79, 150.56, 138.88, 137.10, 122.12, 119.77, 112.52, 67.05, 64.97, 56.02, 46.78, 44.07, 42.22, 36.43, 35.03, 31.45, 27.60, 25.17, 23.20, 15.21.

## Results and discussion

The study started with the preparation of BFMC *via* the reaction of vanillin with CO_2_ derived bis(trichloromethyl)carbonate (BTC), which is a more safer reagent than toxic phosgene,^23^ and the crude product could be easily purified by recrystallization from dichloromethane/hexane achieving a yield of 93%, presenting good potential for scaling up preparation in the future. Further reduction of BFMC by using NaBH_4_ produced a BHMC monomer with a yield of 74%. Their structure were confirmed by ^1^H NMR, ^13^C NMR, and FTIR, as shown in Fig. S1–5.†

Dialdehyde monomers have been used as polymeric monomers in polymer synthesis *via* aldol reaction,^24^ Tishchenko reaction^25,26^ and Baylis–Hillman reaction.^27,28^ In recent years, the multicomponent reactions (MCRs) have been widely applied in the field of synthetic polymers to obtain sequence-regulated polymers, which turn out to be an efficient way to regulate the polymer properties by selecting various structural monomers.^29–34^ For example, Meier firstly employed the Passerini 3CR together with olefin metathesis to afford high molecular weight polyesters with amide moieties in side chain from renewable resources, which have been used for various polymers synthesis recently.^35,36^ Bearing the dialdehyde functional moiety of BFMC, the BFMC monomer was submitted to Passerini 3CR together with potential biomass-derived succinic acid (SA),^37^ adipic acid (AA)^38^ and *tert*-butyl isocyanide derived from glycine under mild conditions, achieving a new class of poly(carbonate ester)s oligomers with amide moieties in their side chain. As shown in Table 1, the yield increased from 71.2% to 76.7% as the reaction time increased from 24 h to 36 h, but further increasing the reaction time to 48 h did not result in an increasing in the yield (Table 1, runs 1–3). The molecular weight were determined by GPC and the results were summarized in Table 1. As the BFMC and AA have fairly good solubility in DCM, it was then used as the polymerization media (Table 1, run 5). Compared with the PCEA-2a obtained in acetonitrile (Table 1, run 4), the molecular weight of PCEA-2b prepared in DCM was increased by 15%, from 4.6 to 5.3 kg mol^−1^; the yield was enhanced to 94.8% as well.

**Table 1:** Multicomponent polymerization based on BFMC^a^, polycondensation of BHMC with diacyl chlorides^b^ and polyaddition of BHMC with diisocyanates^c^

Run	Sample	Monomer	Diacid/acyl chloride/diisocyanate	*T* (°C)	Time (h)	Yield^d^ (%)	*M*_n_ e (g mol^−1^)	PDI^e^ (*M*_w_/*M*_n_)	*T*_g_ (DSC) (°C)	*T*_d_ (5%) (°C)
1	PCEA-1a	BFMC	SA	40	24	71.2	3400	1.4	106	185
2	PCEA-1b	BFMC	SA	40	36	76.7	4700	1.7	102	206
3	PCEA-1c	BFMC	SA	40	48	77.3	4700	1.5	93	210
4	PCEA-2a	BFMC	AA	40	24	73.6	4600	1.6	71	225
5^f^	PCEA-2b	BFMC	AA	40	36	94.8	5300	1.3	86	235
6	PCE-1a	BHMC	SC	25	24	82.4	5800	1.7	37	224
7^g^	PCE-1b	BHMC	SC	25	24	78.1	7900	1.6	54	255
8	PCE-2a	BHMC	TC	25	24	71.9	3100	1.3	74	256
9	PCU-1a	BHMC	MDI	25	24	68.7	15 900	1.8	97	130
10	PCU-1b	BHMC	MDI	40	24	73.4	16 400	1.4	106	161
11	PCU-2a	BHMC	IPDI	25	24	62.1	19 800	2.2	121	146
12	PCU-2b	BHMC	IPDI	40	24	67.5	24 400	1.6	138	166

a Conditions: BFMC (1 mmol), diacid (1 mmol) and *tert*-butyl isocyanide (2 mmol), acetonitrile as solvent and N_2_ purged.

b Conditions: BHMC (1.0 mmol), diacyl chloride (1.0 mmol), pyridine (2.2 mmol), THF as solvent and N_2_ purged.

c Conditions: BHMC (1 mmol), diisocyanate (1.0 mmol), DBU (3%mol), THF as solvent and N_2_ purged.

d Isolated yield *via* precipitation into excess diethyl ether and vacuum dryness (50 °C, 24 h).

e Determined by a gel permeation chromatograph (GPC) in THF relative to PMMA standards.

f DCM was used as solvent.

g TEA (2.2 mmol) was used as the base.

**Fig. 1:**
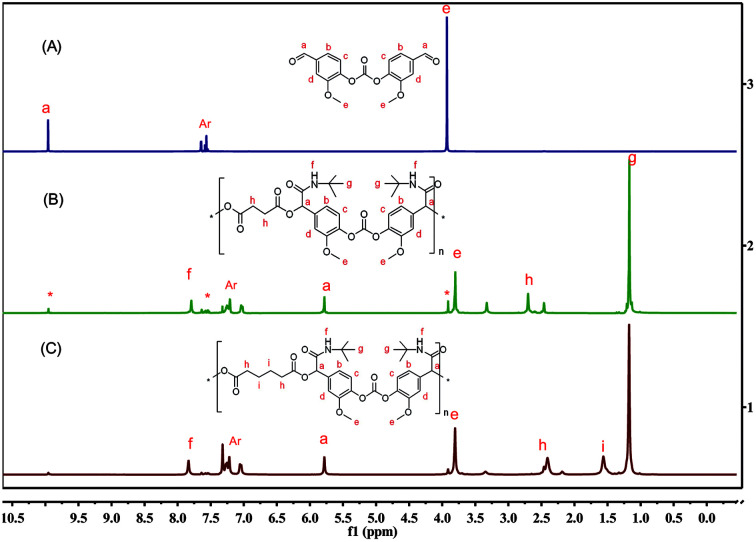
Overlay of ^1^H NMR spectra (DMSO-d_6_) of BFMC (A) and PCEAs obtained from BFMC (B and C),* for terminal aldehyde group.

The structure of PCEAs were confirmed by NMR and FTIR. As shown by the overlay of the ^1^H NMR spectra of BFMC and PCEA-1 in Fig. 1, the resonance of the aldehyde group of BFMC at 9.97 ppm almost disappears, while new sharp peaks are observed at 7.83 and 2.74 ppm, which are attributed to the amide group and the methene moiety. FTIR spectra (Fig. S10†) show the characteristic changes of the functional groups. It is apparent that new absorption band at 3398 cm^−1^ and 1744 cm^−1^ (stretching vibration of the amide group –NH–CO–) were observed. The sequential information (*i.e.*, carbonic ester-amide sequence) formed from MCRs can be incorporated into the structure of the polymer backbone at the same time. The thermal properties of PCEAs were also examined by differential scanning calorimetry (DSC) and thermal gravimetric analysis (TGA; Fig. S11 and S12†). PCEAs can be stable up to 235 °C depending on their molecular weight, above which slow decomposition occurred.

The onset decomposition temperature of PCEAs increases with increase in the molecular weight. The two stage decomposition occurs which may be caused by the decomposition of ester bond and carbonated linkage. PCEAs are amorphous and exhibit *T*_g_s ranging from 70 to 106 °C, and no melting temperature are detected. PCEAs have been widely used in many industries such as fibers, biodegradable materials, plastic films *etc.*,^39,40^ owing to the combined advantages of polyester and polyurethane. The as-prepared polymers are a kind of copolymer that contains both carbonate bonds (–O–CO–O–) and amide bonds (–CO–NH–) on the macromolecular chain. If functional isonitriles are used, functional side groups can be easily introduced at the same time, providing a simple method for further modification towards desirable properties.

**Fig. 2:**
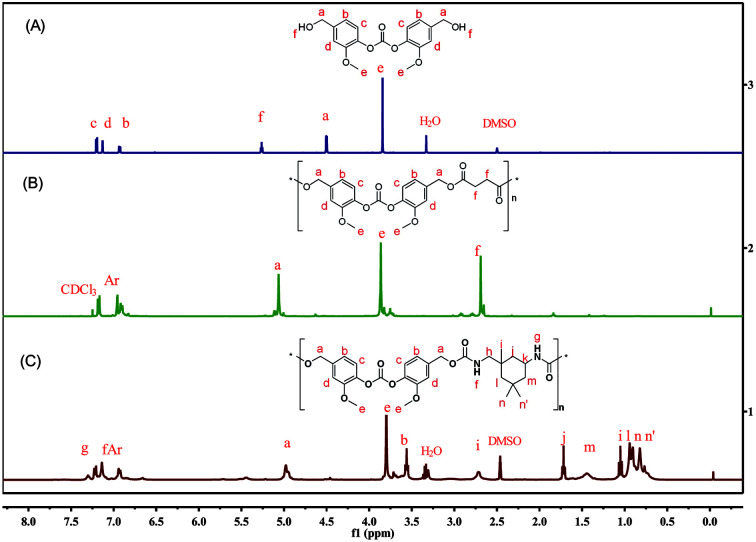
Overlay of ^1^H NMR spectra of BHMC (A) as well as PCE-1 (B) and PCU-2 (C) prepared from BHMC.

The BHMC monomer was firstly applied in the polycondensation with diacyl chlorides including both potential biomass-derived succinyl chloride (SC)^41^ and terephthaloyl dichloride (TC) in THF.^42,43^ For SC, the isolated yield of PCE-1a was up to 82.4% and the molecular weight was 5.8 kg mol^−1^ (Table 1, run 6), while PCE-2 derived from TC shown similar yield of 71.9% but with a lower *M*_n_ of 3.1 kg mol^−1^ (Table 1, run 8). The polydispersity indexes (PDIs) between 1.3 and 1.7. The NMR and FTIR were used to elucidate the chemical structures of the as-prepared PCEs, and the results were shown in Fig. 2A and B.

The ^1^H NMR of BHMC was also enclosed towards a comparative purpose. The newly formed peak at 2.70 ppm in ^1^H spectra is attributed to the saturated succinyl moiety. In the ^13^C NMR spectra (Fig. S16†), the carbonyl carbons of the newly formed ester linkage in the PCE-1 appears at 172.09 ppm, while the ester linkages from the carbonate moieties is observed at 150.60 ppm, similar to the values observed in the monomer (150.45 ppm). In the FTIR spectra (Fig. S19†), the broad absorption peak at 3390 cm^−1^ is the stretching vibration of the hydroxyl groups (–OH) of BHMC. The strong absorption peak centred at 1770 cm^−1^ is attributed to the stretching vibration of carbonyl groups in carbonates. After the polymerization, new strong absorption peaks appears at about 1734 and 1115 cm^−1^, due to the newly formed carbonyl group (C=O) and ether group (C–O–C) in the ester linkage (C–O–CO). The results of NMR and FTIR confirm the successful preparation of PCEs.

The DSC curves of these PCEs (Fig. S20†) show that, PCE-1b bearing a longer soft segment [–(CH_2_)_2_–] has a lower *T*_g_ of 37 °C than that (*T*_g_: 74 °C) of PCE-2a containing a rigid segment (–Ph–) in the main chain. The onset decomposition temperatures (5% weight loss) of all the PCEs are above 220 °C, and the PCE-2a has the highest *T*_d_ of 256 °C (Fig. S21†). Comparatively, although PCEs have similar main chain structures, the poly(carbonate ester) containing amide group side chain showed higher *T*_g_ but lower thermal stability.

**Scheme 1:**
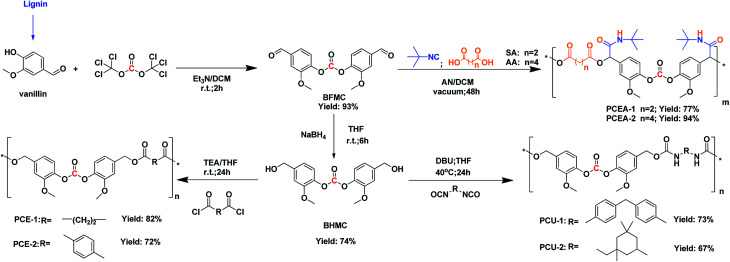
The design and synthesis of polymeric monomers and their polymers.

As one of the most important categories of polymeric materials, PUs are widely used as coatings, adhesives, sealants, foams, *etc.*,^44–47^ due to their versatile properties determined by their constitutional unit and topological structure.^48^ Compared with traditional polyether PUs and polyester PUs, PCUs are characterized by a large amount of carbonate structure in the main chain, which gives poly(carbonate urethane)s unique properties beyond the polycarbonates or polyurethanes.^49–51^ Traditionally, the preparation of PUs involved the polyaddition reaction of polyisocyanates and polyols.^52^ It is well accepted that the use of biorenewable polyols is anticipated to increase the environmental and sustainable issues of PU.^53^ Therefore, polyadditions of BHMC with diisocyanates (Scheme 1), including diphenylmethane diisocyanate (MDI) and isophorone diisocyanate (IPDI), were conducted in THF using 1,8-diazabicyclo[5.4.0]undec-7-ene (DBU) (3 mol%) as catalyst, achieving a pale yellow solid PCUs. Unlike the polycondensation of BHMC with diacid chlorides, this polyaddition with diisocyanates gives PCUs with higher molecular weights (*M*_n_ = 15.9–24.4 kg mol^−1^) and dispersity (PDIs = 1.36–2.17) (Table 1, runs 9–12). In addition, increasing the temperature is beneficial to the yields and the molecular weight, but the excessively high temperature may cause the gel effect to be detrimental to the experimental results.

^1^H NMR and ^13^C NMR were employed to confirm the structures of the as prepared PCUs. Taking the ^1^H NMR spectrum of PCU-1 as an example (Fig. S26†), there were no peaks for the hydroxyl group at around 5.26 ppm, while the typical chemical shifts at 9.72 ppm is observed, which are assigned to the amide proton of the newly produced urethane linkage [–OC(=O)–NH–]. In the ^13^C NMR spectrum of PCU-1 the chemical shifts at 153.35 ppm can be assigned to the newly formed carbons of amino linkage (Fig. S27†). Similar results can be found in the ^1^H NMR and ^13^C NMR spectra of as prepared PCUs from IPDI (Fig. 2C and S29†). The formation of these PUs were further confirmed by FTIR (Fig. S30†). The broad stretching vibration absorption band of O–H (3390 cm^−1^) in BHMC is replaced by the N–H (3400 cm^−1^) band in the prepared PCUs. Such urethane linkages are further evidenced by the appearance of a new strong peak at 1718 cm^−1^, assigning to the newly formed carbonyl C=O stretching vibration absorption.

**Fig. 3:**
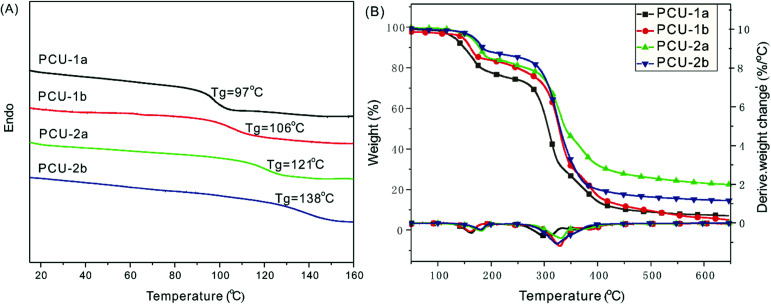
DSC curves (A) and TGA traces (B) of PCUs.

The TGA traces (Fig. 3B) indicate that these PCUs display two apparent stages of thermal decomposition accompanied by two maximum rate decomposition temperatures. The first stage, where the PCU weight lost slowly (weight loss: around 20% for PCU-1; 15% for PCU-2), is attributed to the rupture of the CO–NH urethane bonds, and the second stage is due to the pyrolysis of the [–O–C(=O)–O–] and C–C bond. The onset decomposition temperatures (5% weight loss) for the PUs in the range of 130–166 °C. Usually, the MDI-based polyurethane has higher *T*_g_ and thermostability than those of the IPDI-based polyurethane due to its aromatic feature.^54^ However, the IPDI- based PCU-2b displays a *T*_g_ of 138 °C, whereas it is only 106 °C in the case of the rigid MDI-based PCU-1b (Fig. 3A), and the reason is still unclear at the current stage of the study.

Bearing the potential renewable origin of vanillin, SA and adipic acid in mind, the as-prepared PCEAs and PCEs polymers present good examples for the design and synthesis of polymers with low carbon footprint. The E-factor analysis was employed to evaluate the environment impact of our approach and the details are shown in Tables S1–S6.† According to our calculations, the E-factor of vanillin-based polymer PCEA-2b, PCE-1b and PCU-1b were determined as 4.350, 12.1 and 8.976 kg kg^−1^, respectively, which are in accordance with Sheldon's analysis of bulk and fine chemicals having an E-factor of 4–50.^55–57^

## Conclusions

In conclusion, novel symmetric BFMC and BHMC polymeric monomers have been synthesized in high yields using vanillin and CO_2_ derived BTC as raw chemicals. Their potential in sustainable polymer synthesis have been identified by the successful synthesis of novel classes of poly(carbonate ester)s oligomers, poly(carbonate ester)s pending amide moiety oligomers, and poly(carbonate urethane)s. The structure and thermochemical properties have been elucidated and evaluated by various characteristic technologies that should open interesting possibilities for application due to the particular structure of them. It is also important to note that the prepared PCEA and PCE are mainly of renewable origin and might thus contribute to a sustainable development. The findings provide important insight for the design and preparation of new bio-based synthetic polymers.

## Conflicts of interest

The authors declare no competing financial interest.

## Supplementary Material

RA-008-C8RA07185C-s001
