# Supplementary material for: Vanillin derived a carbonate dialdehyde and a carbonate diol: novel platform monomers for sustainable polymers synthesis
Source: RSC Adv. 2018 Oct 5;8(60):34297–303. doi: 10.1039/c8ra07185c (PMC9087012; doi:10.1039/c8ra07185c)
Supplement: RA-008-C8RA07185C-s001 [file RA-008-C8RA07185C-s001.pdf]

Electronic Supplementary Material (ESI) for RSC Advances.
This journal is © The Royal Society of Chemistry 2018

Supporting Information

Vanillin derived a carbonate dialdehyde and a carbonate diol: novel platform monomers for sustainable polymers synthesis

De Bai,[a]# Qin Chen,[a]# Yang Chai,[a] Tianhua Ren,[a] Caijuan Huang,[a] Qiang Zheng,[a] Ian D. V. Ingram,[b] Michael North[b] and Haibo Xie*[a]

[a] Department of Polymeric Materials & Engineering, College of Materials & Metallurgy, Guizhou University, West Campus, Huaxi District, Guiyang, P. R. China, 550025; E-mail:hbxie@gzu.edu.cn

[b] Green Chemistry Centre of Excellence, Department of Chemistry, University of York, York (UK) YO10 5DD

SFigures

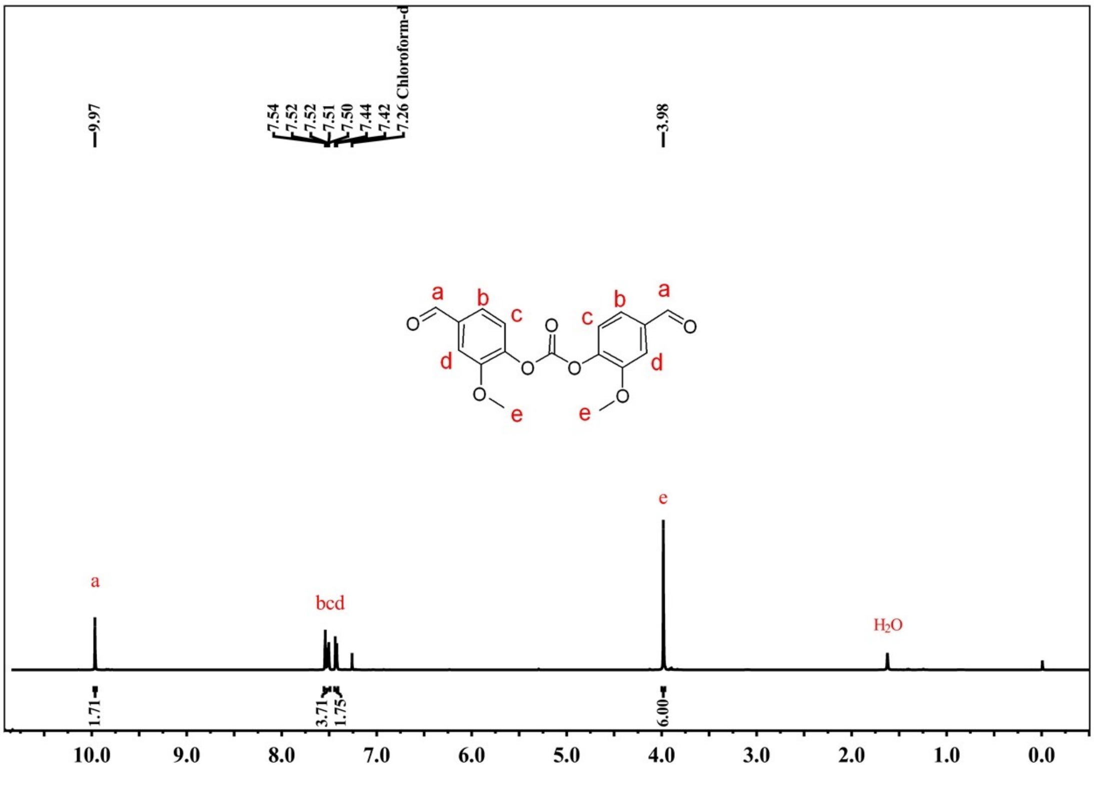

Figure S1. ^{1}H NMR of BFMC (400 MHz $CDCl_3$).

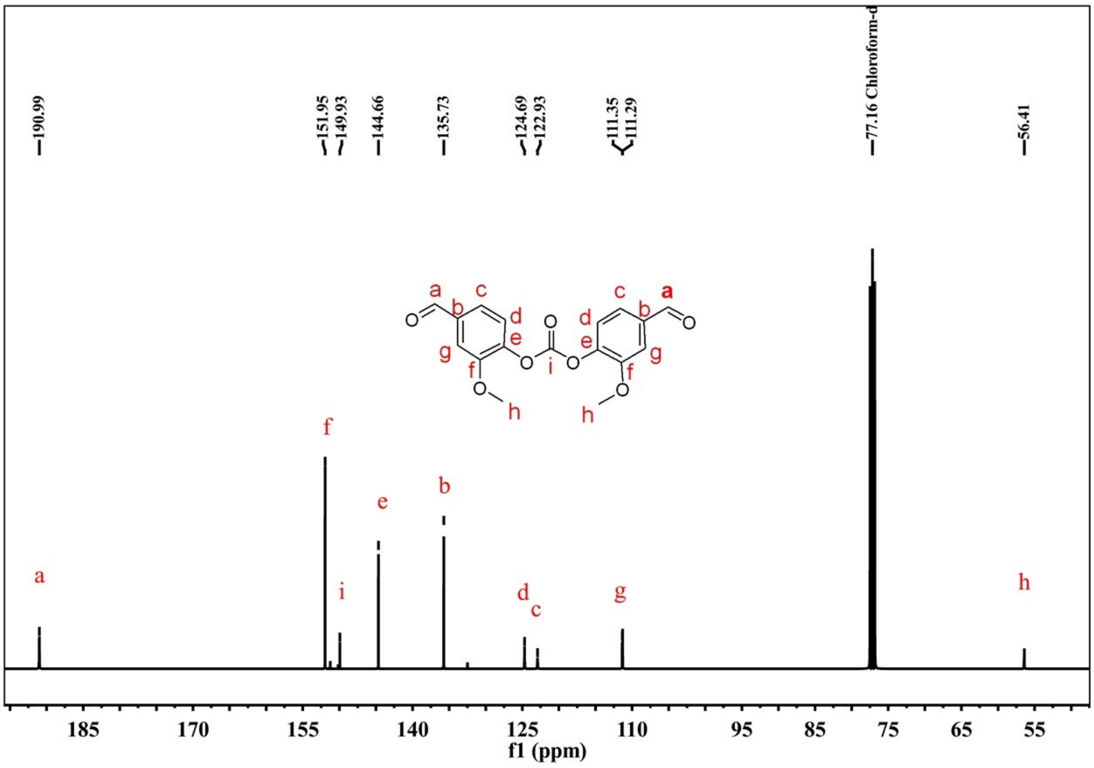

Figure S2. ^{13}C NMR of BFMC (101 MHz $CDCl_3$).

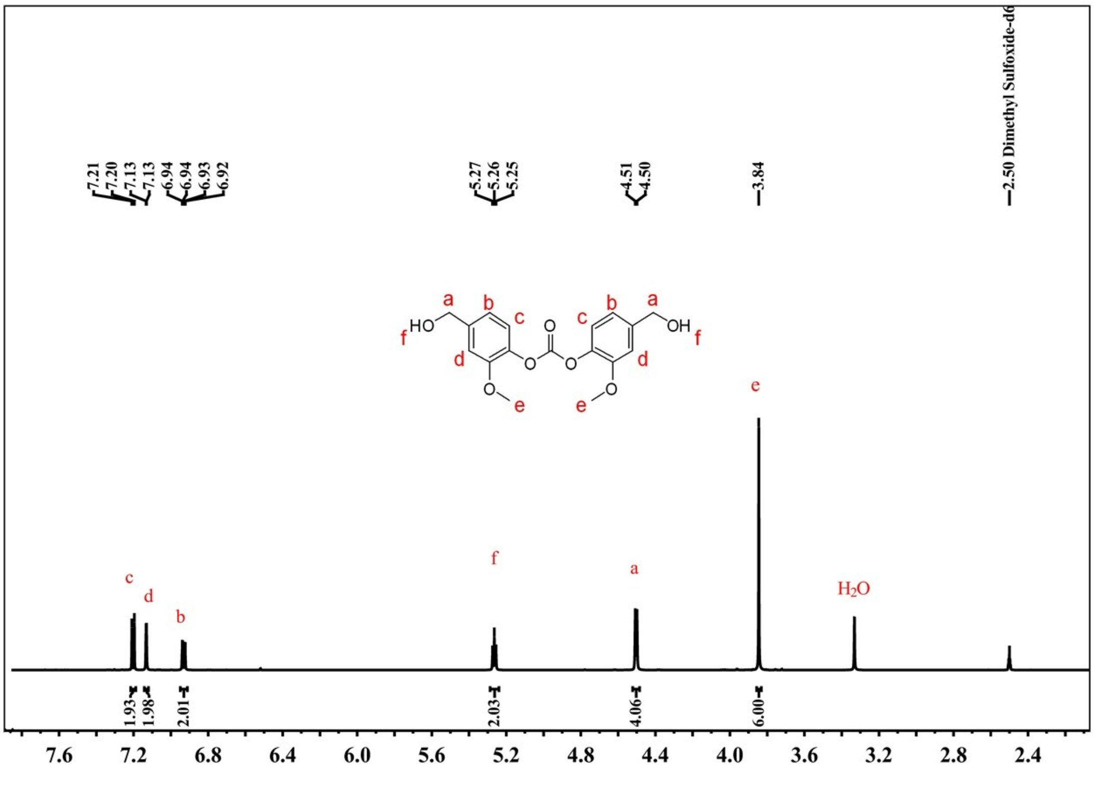

Figure S3. ^{1}H NMR of BHMC (400 MHz DMSO-d_6).

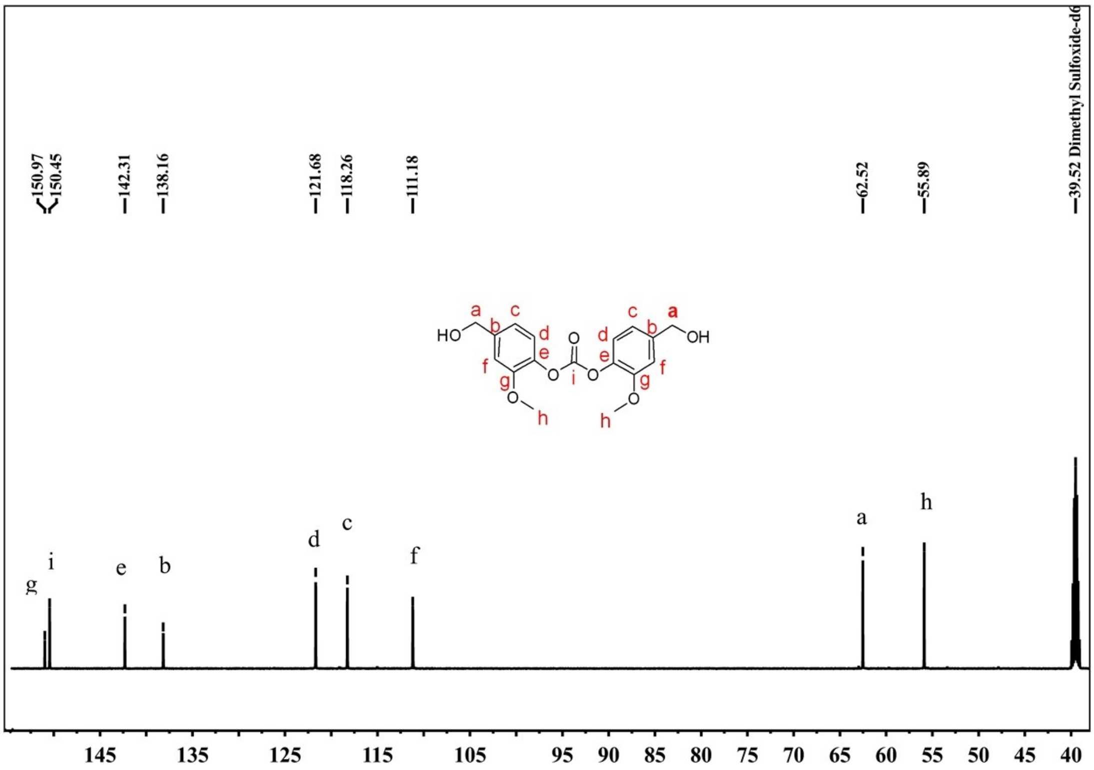

Figure S4. ^{13}C NMR of BHMC (101 MHz DMSO-d_6).

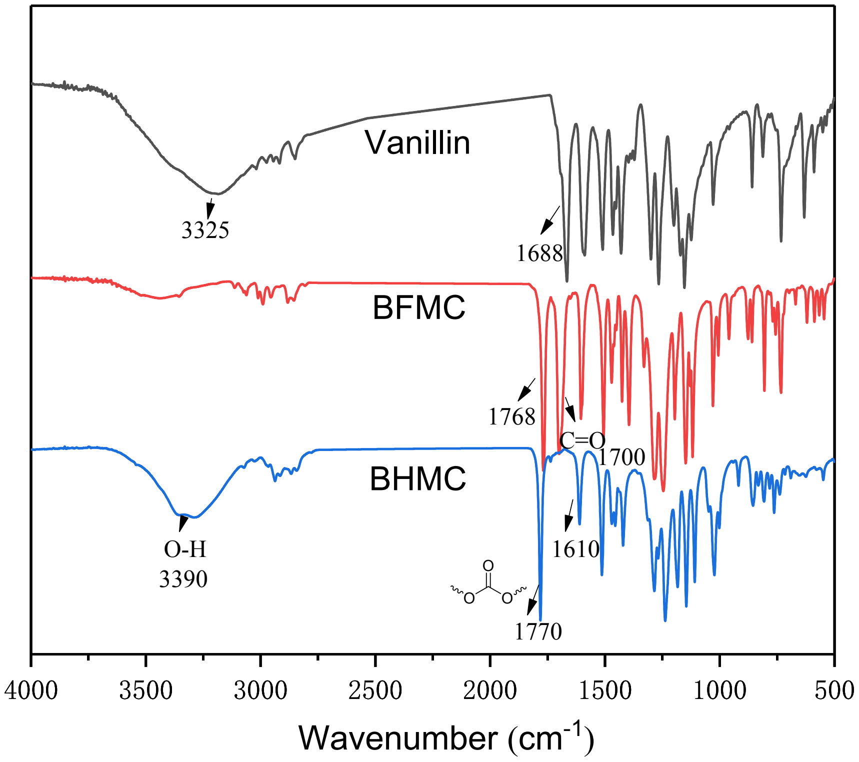

Figure S5. FTIR spectra of BFMC and BHMC derived from vanillin.

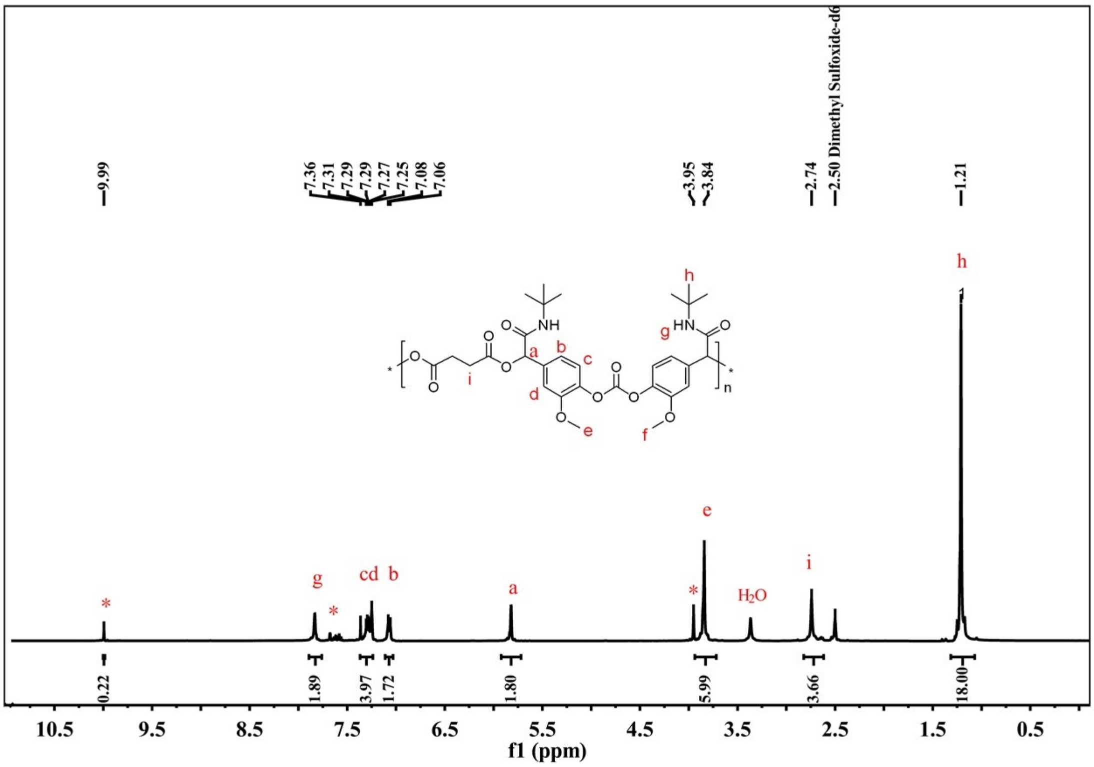

Figure S6. ^{1}H NMR of PCEA-1 (400 MHz DMSO-d_6, *for terminal group).

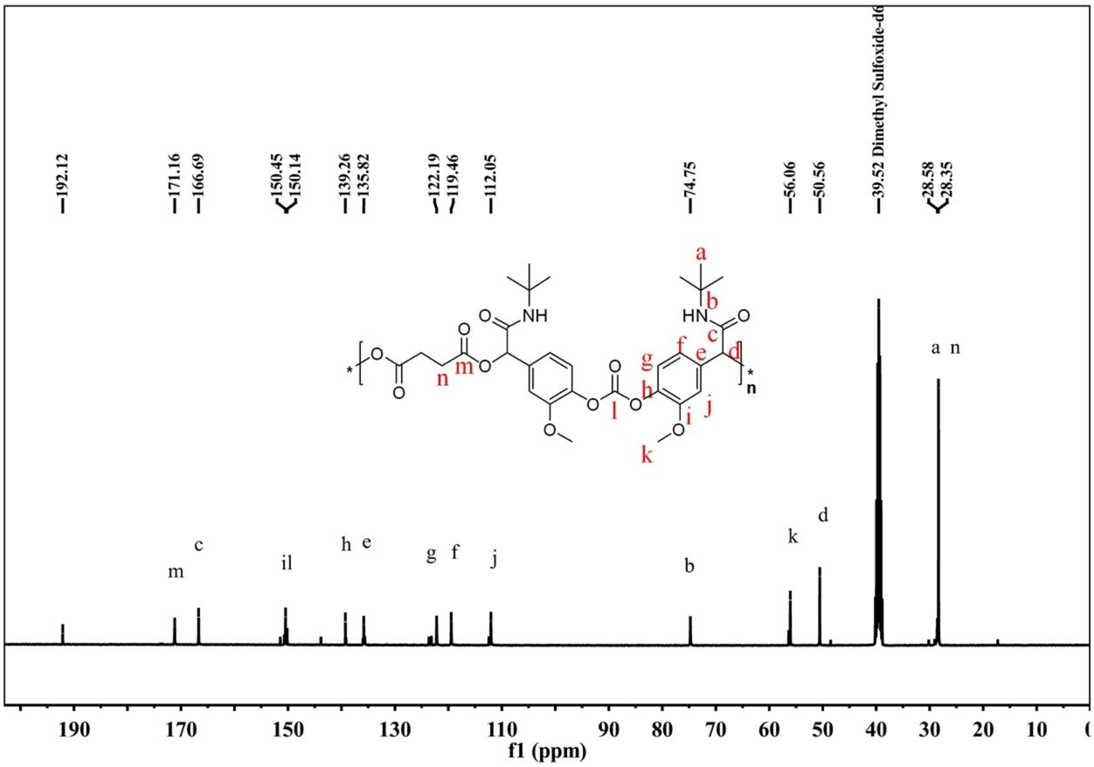

Figure S7. ^{13}C NMR of PCEA-1 (125 MHz DMSO-d_6).

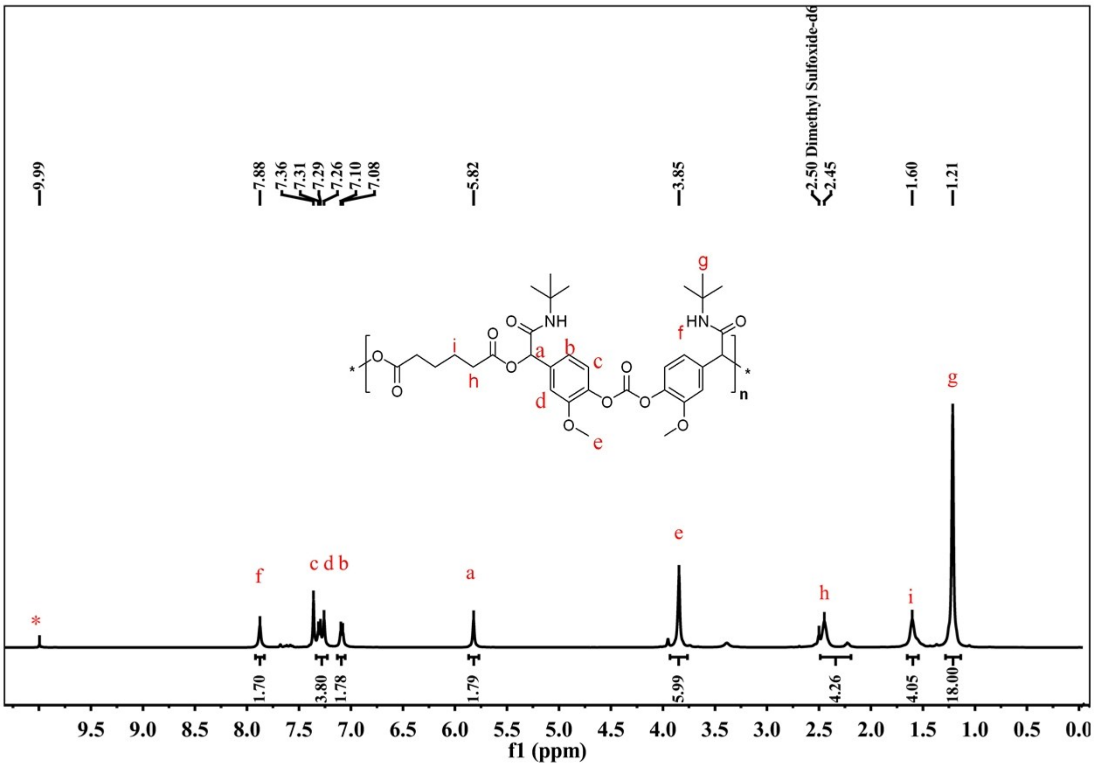

Figure S8. ^{1}H NMR of PCEA-2 (400 MHz DMSO-d_6, *for terminal group).

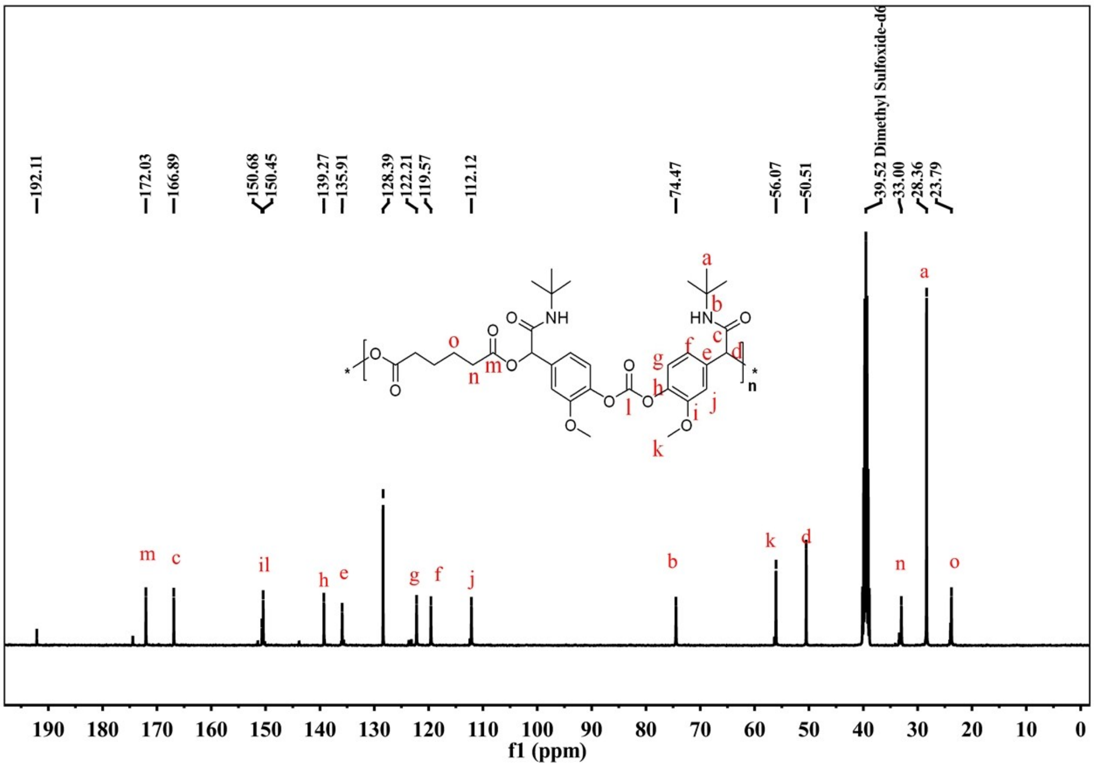

Figure S9. ^{13}C NMR of PCEA-2 (101 MHz DMSO-d_6).

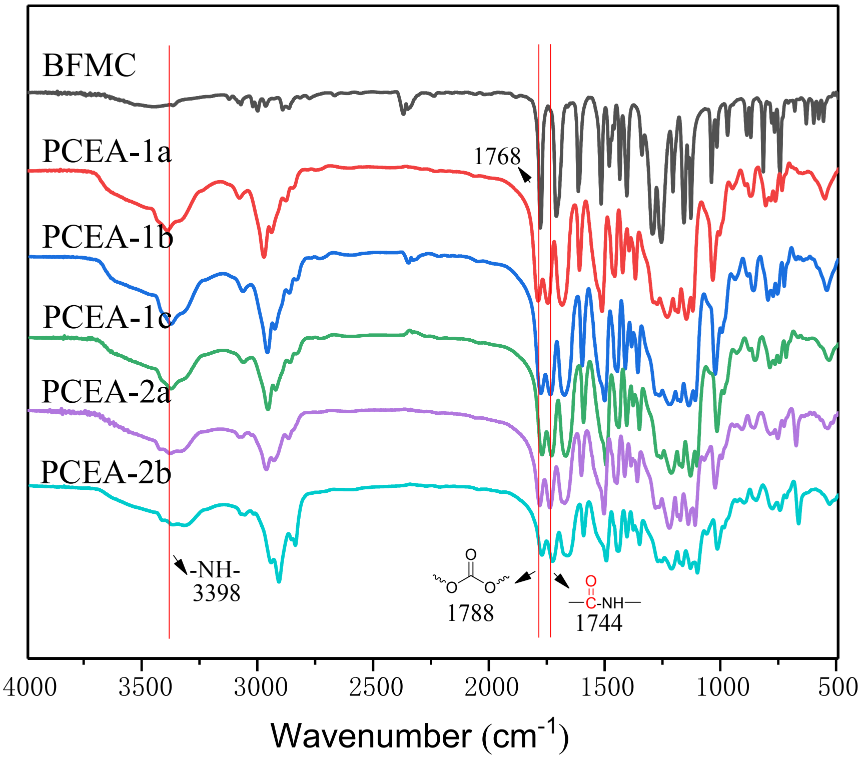

Figure S10. FTIR spectra of BFMC and representative resulting PCEAs.

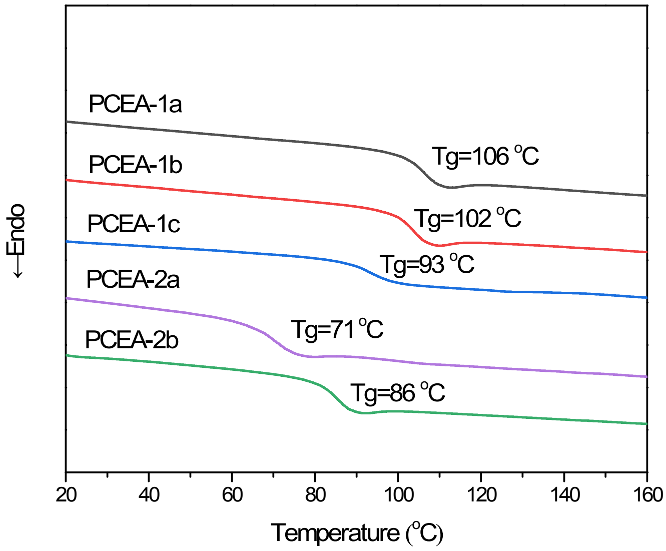

Figure S11. DSC curves of PCEAs.

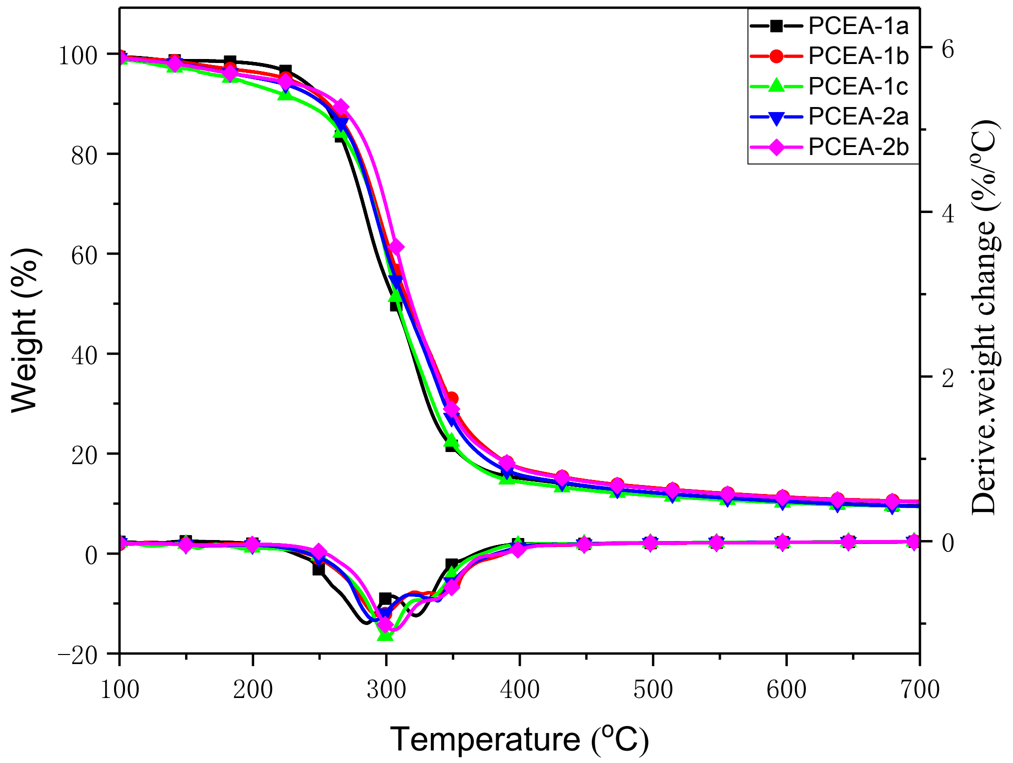

Figure S12. TGA and DTG curves of PCEAs.

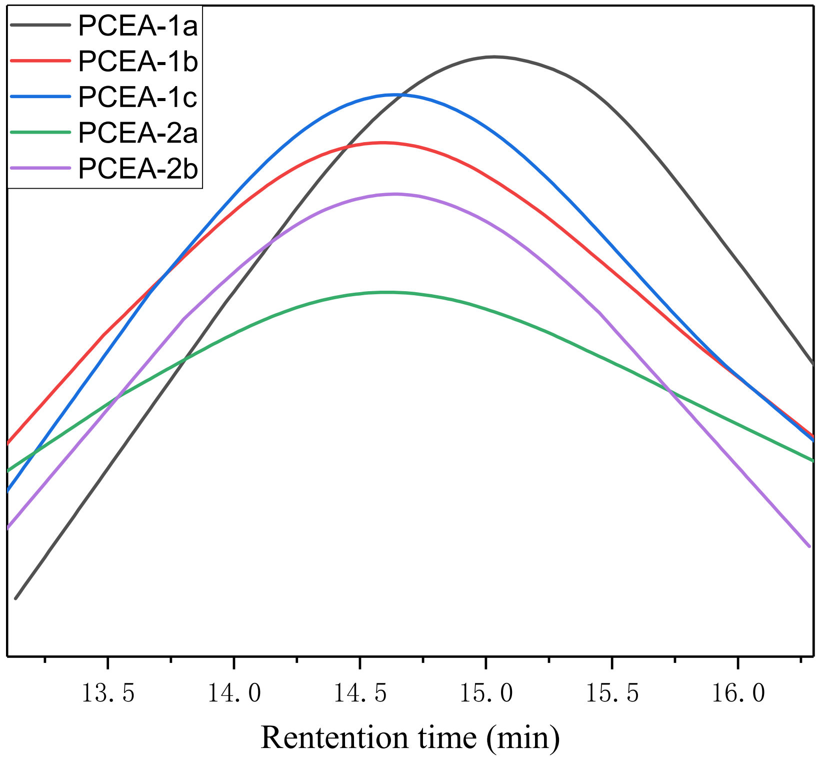

Figure S13. GPC analysis of PCEAs, (PCE-1a, M_n=3456g/mol, Đ=1.4; PCE-1b, M_n=4663g/mol, Đ=1.6; PCE-1c, M_n=4734g/mol, Đ=1.5; PCE-2a, M_n=4575g/mol, Đ=1.6; PCE-2b, M_n=5251g/mol, Đ=1.3.

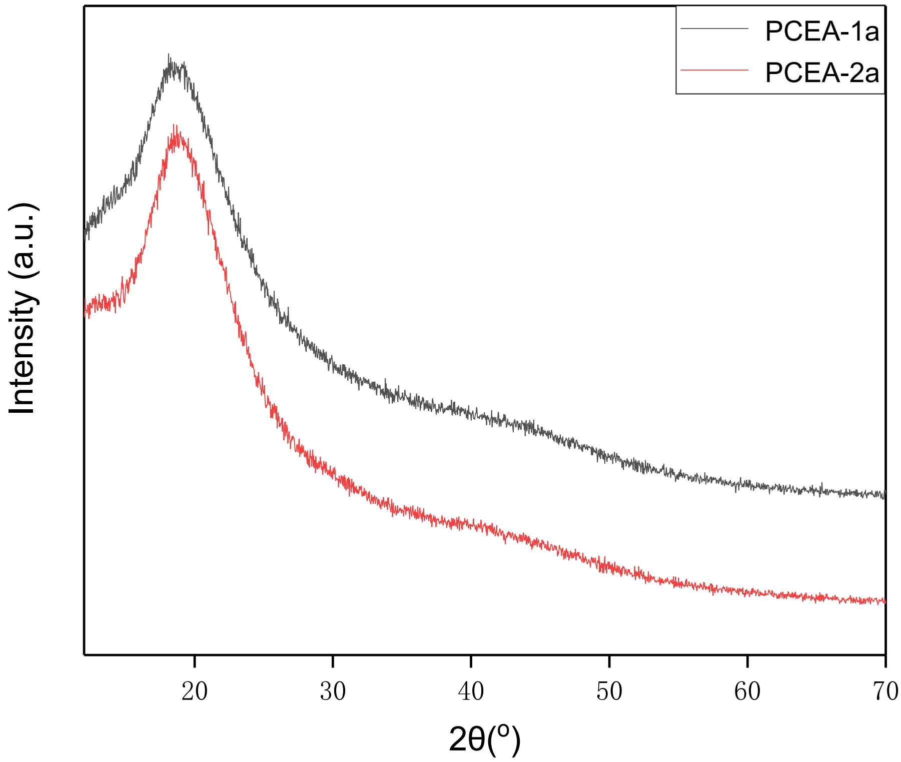

Figure S14. XRD curves of PCEAs.

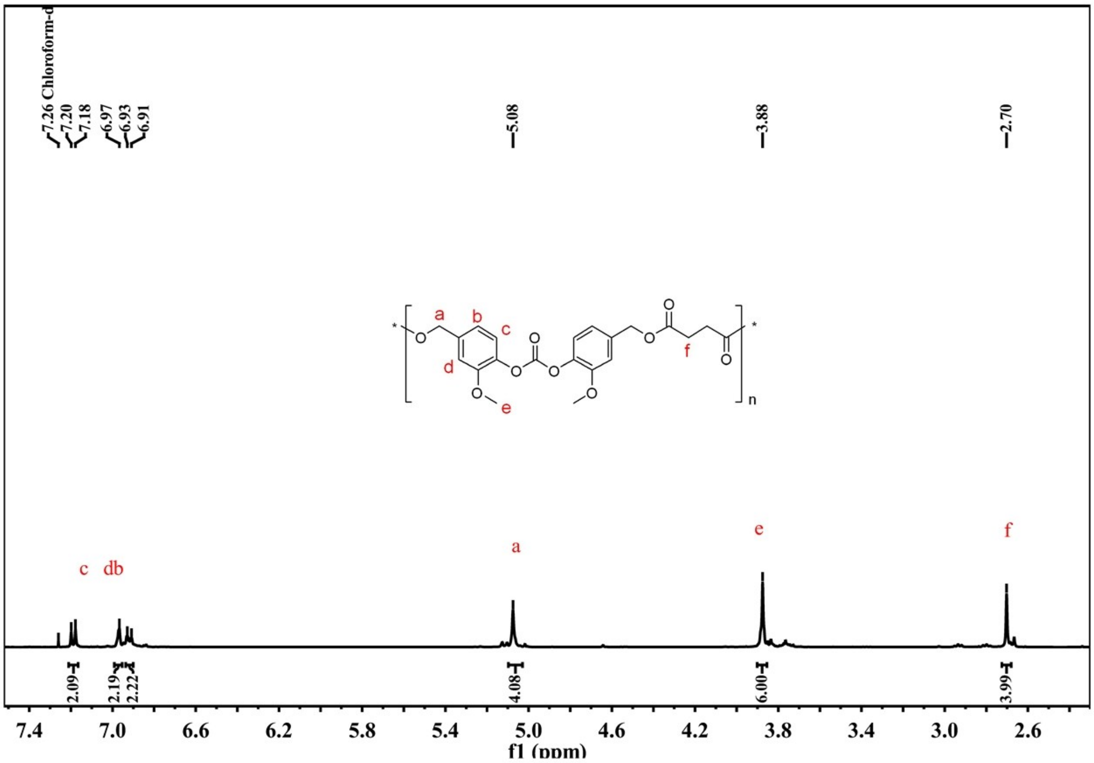

Figure S15. ^{1}H NMR of PCE-1 (400 MHz CDCl3).

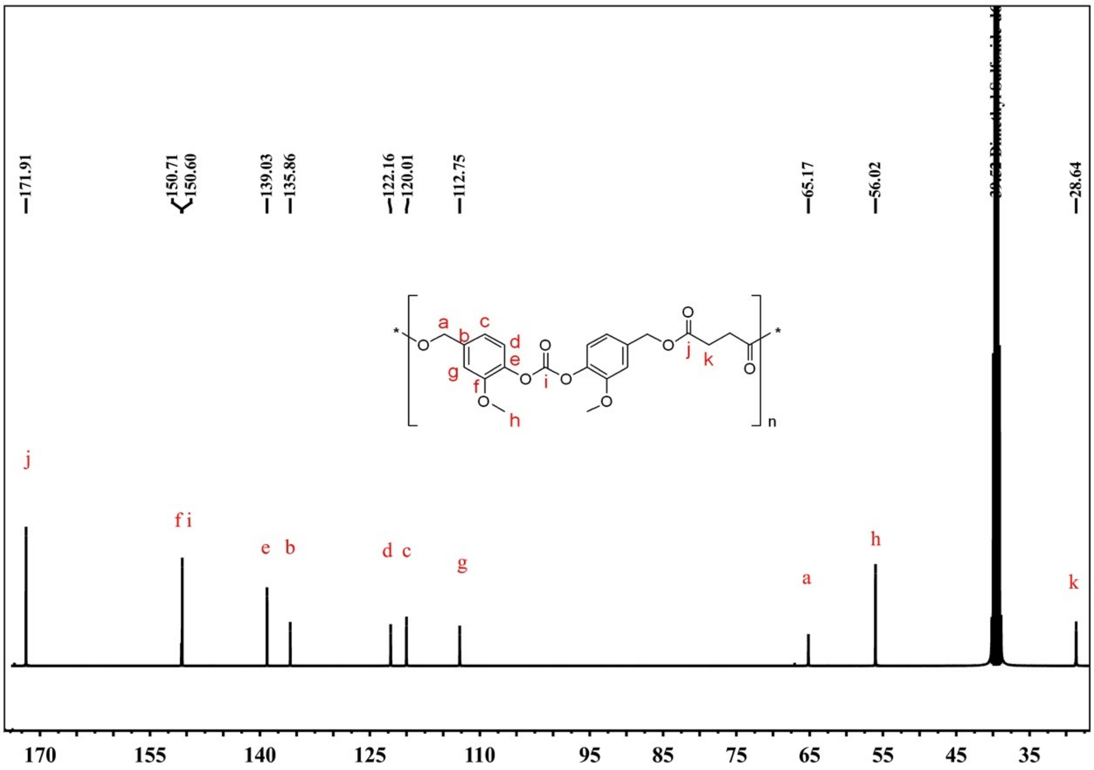

Figure S16. ^{13}C NMR of PCE-1 (101 MHz DMSO-d_6).

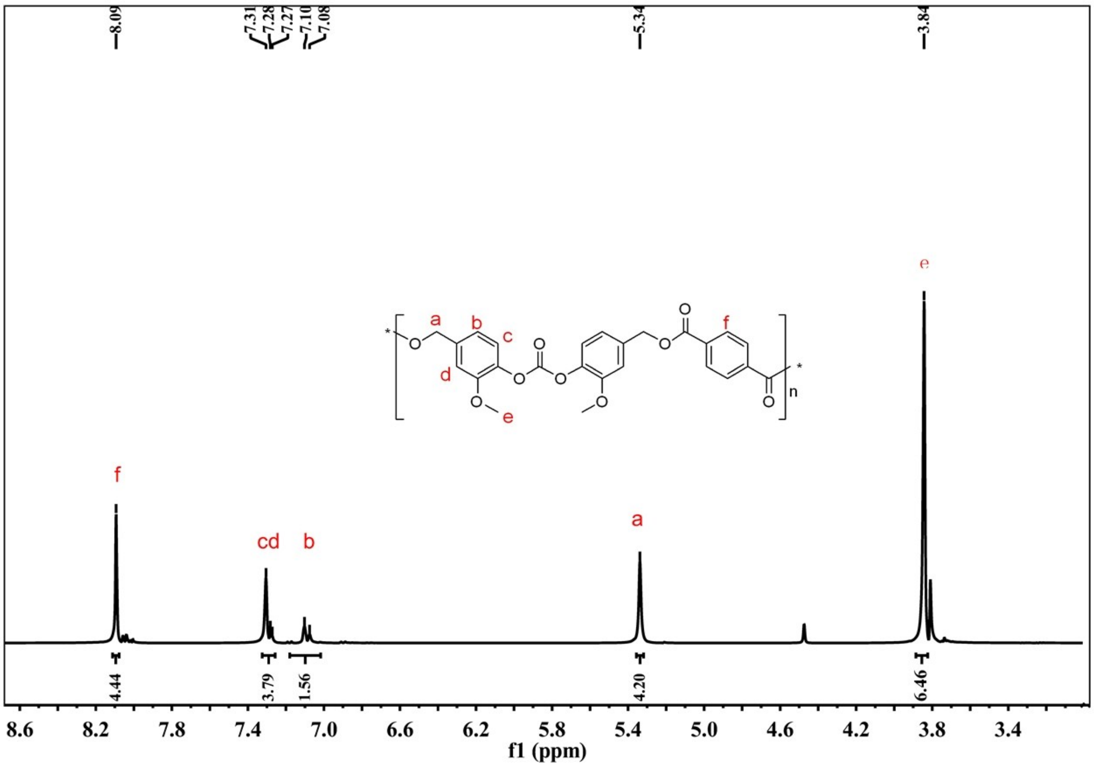

Figure S17. ^{1}H NMR of PCE-2 (400 MHz DMSO-d_6).

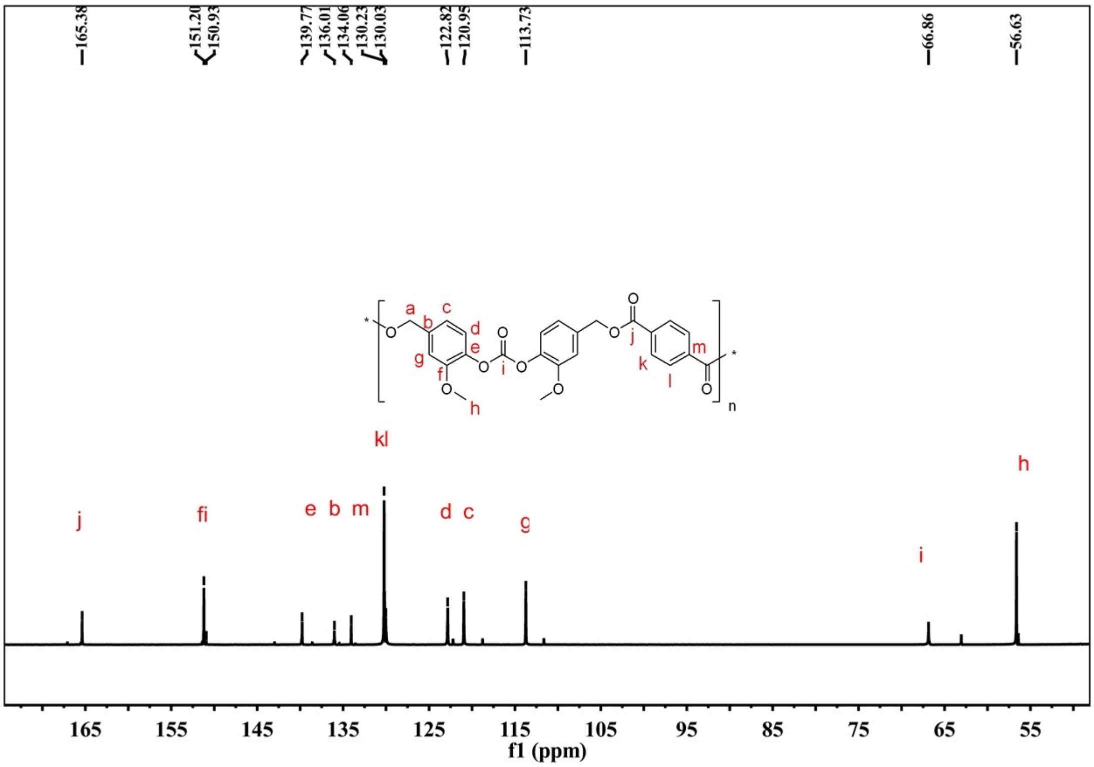

Figure S18. ^{13}C NMR of PCE-2 (101 MHz DMSO-d_6).

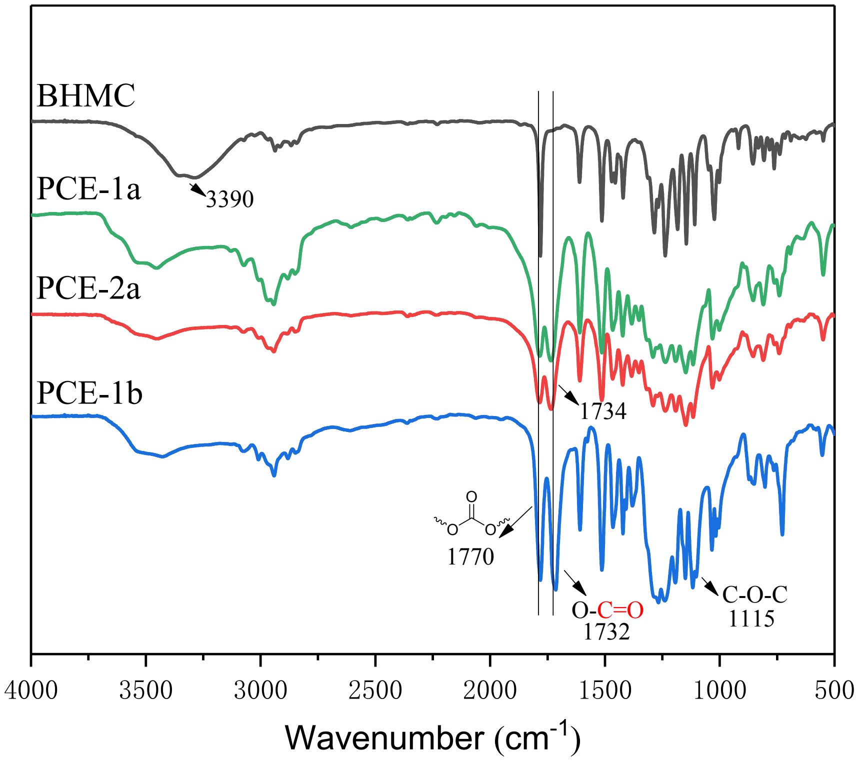

Figure S19. FTIR spectra of BHMC and representative resulting PCEs.

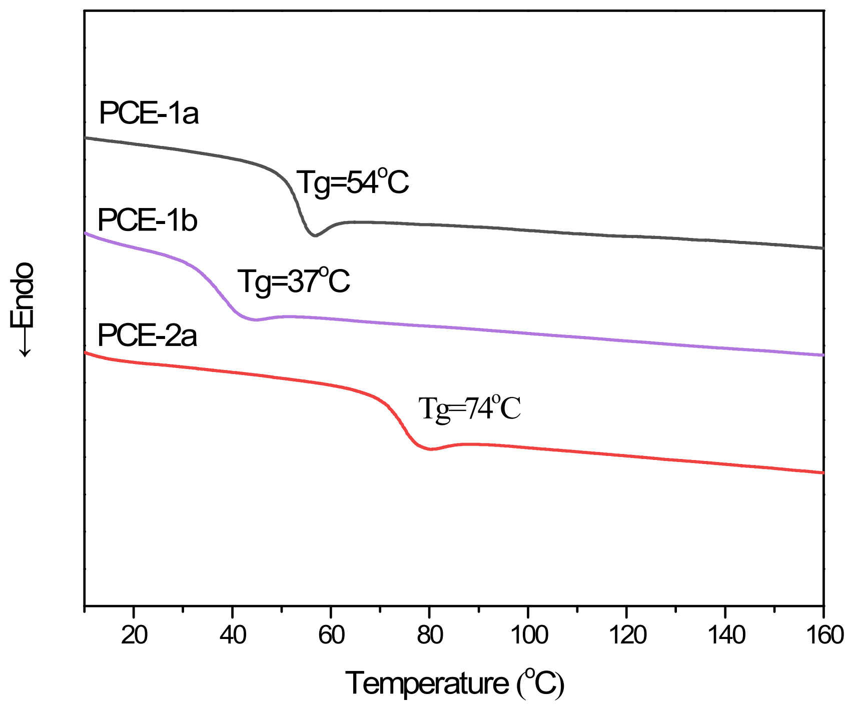

Figure S20. DSC curves of PCEs.

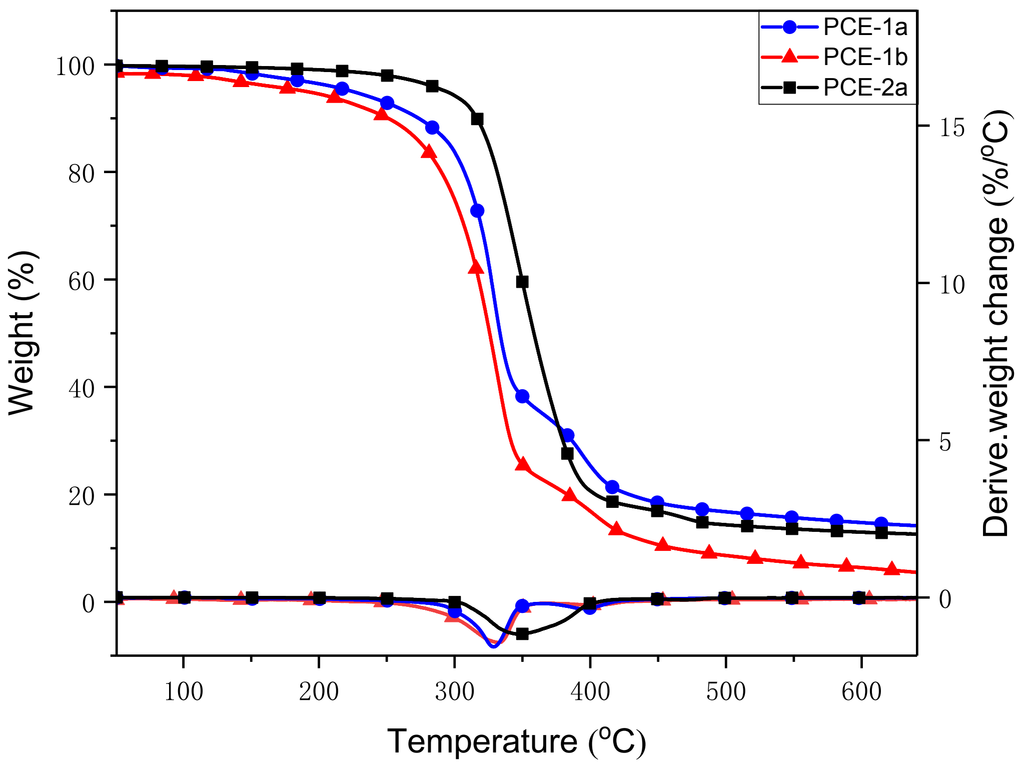

Figure S21. TGA and DTG curves of PCEs.

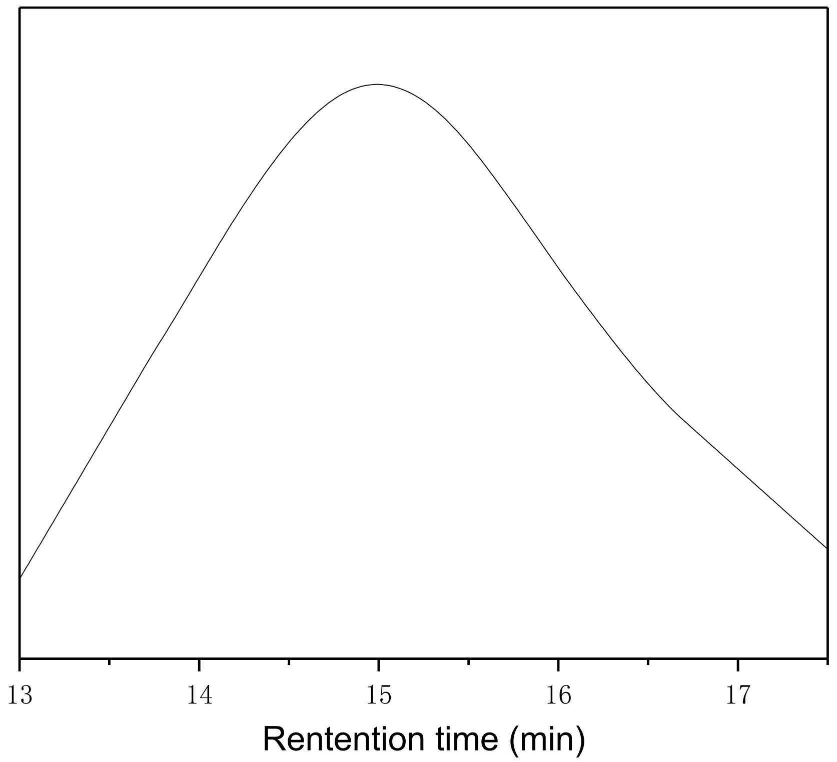

Figure S22. GPC analysis of PCE-1a, M_n=5827 g/mol, PDI=1.65.

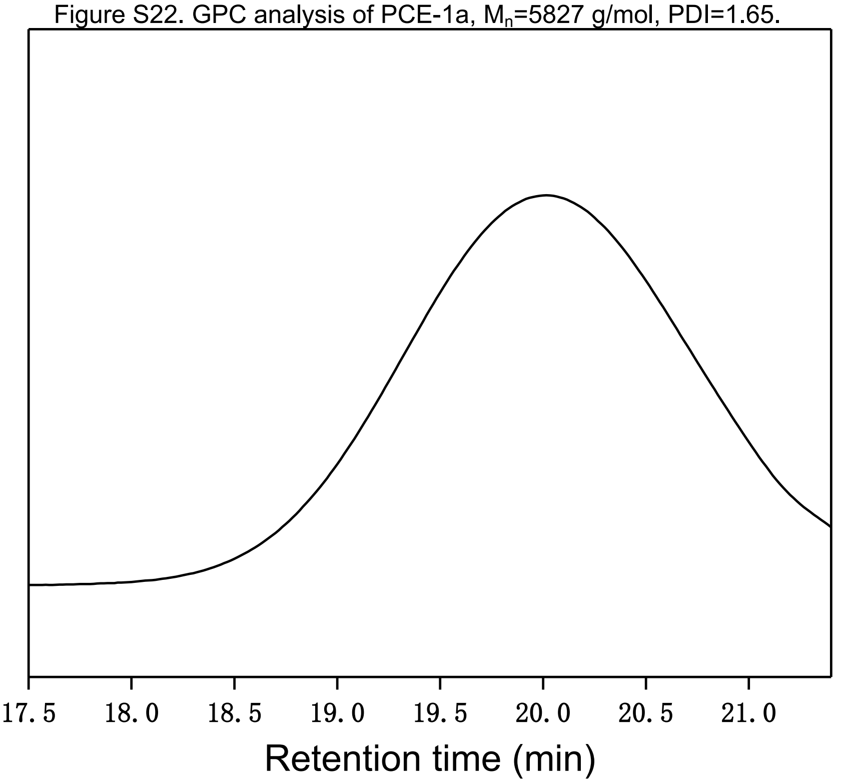

Figure S23. GPC analysis of PCE-1b, M_n=7925 g/mol, PDI=1.61.

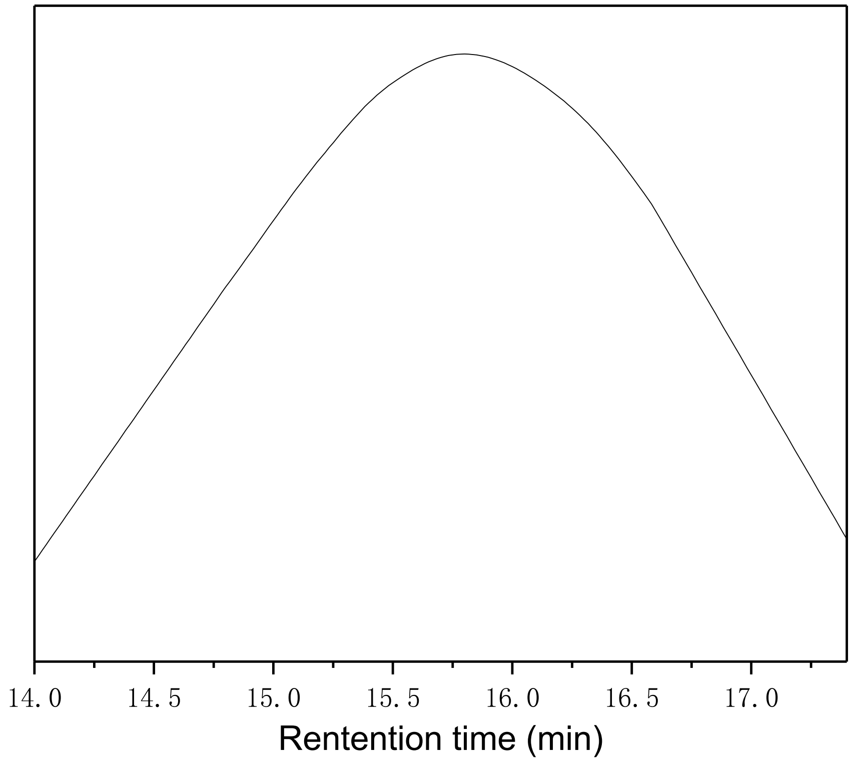

Figure S24. GPC analysis of PCE-2a, M_n=3078 g/mol, PDI=1.31.

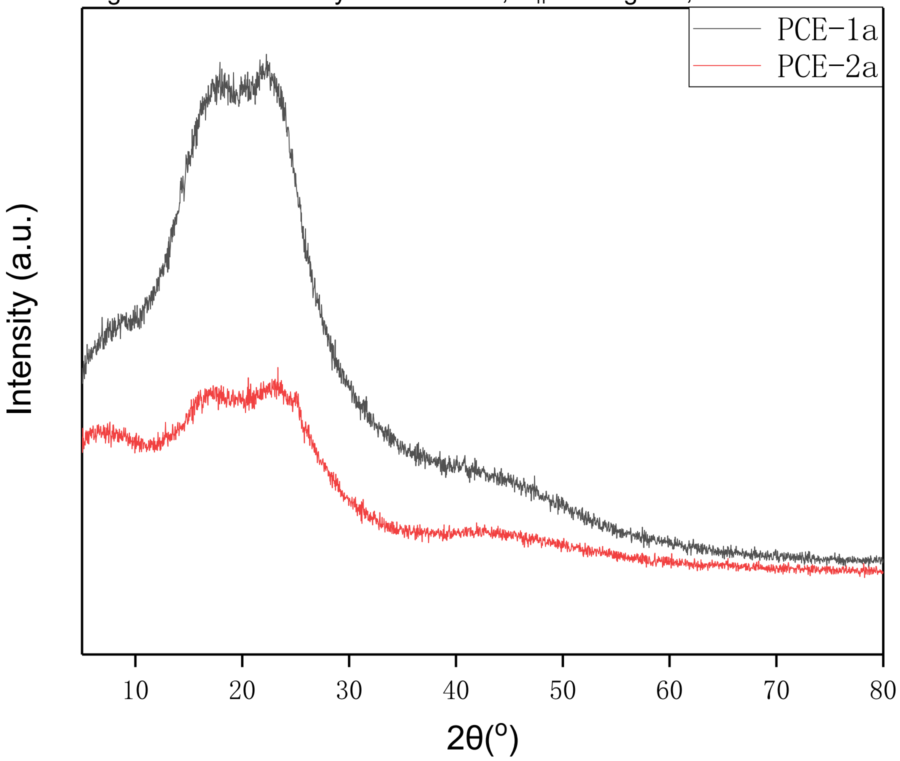

Figure S25. XRD curves of PCEs.

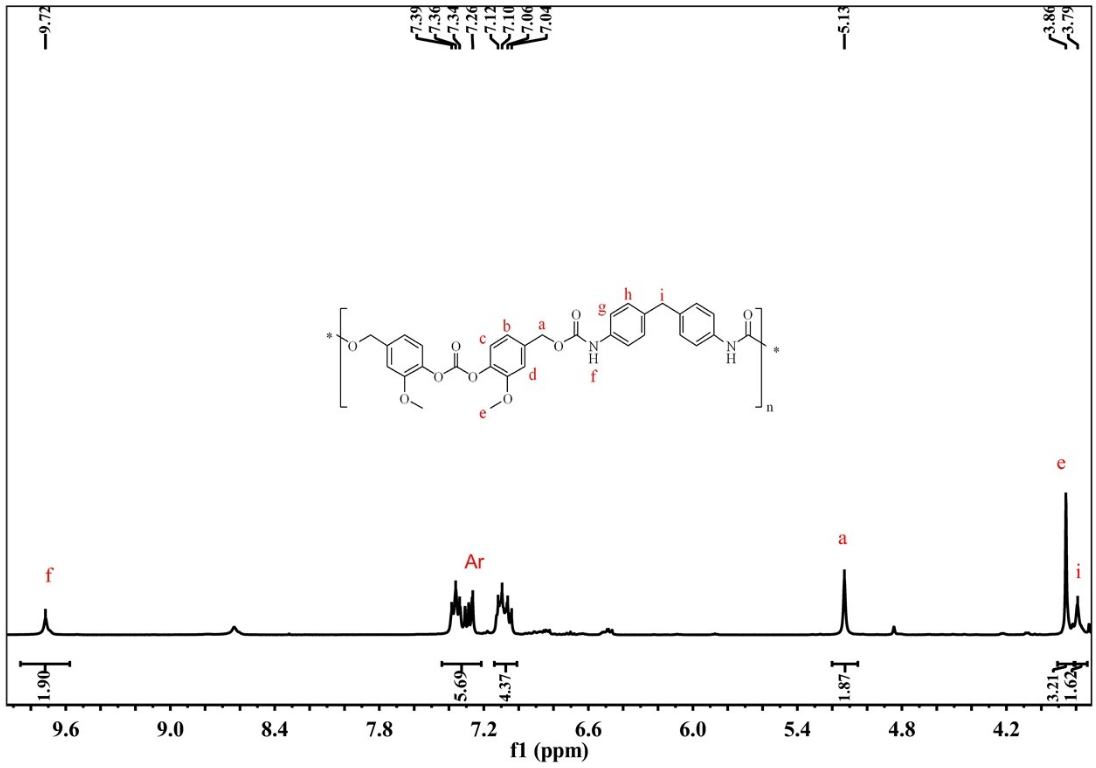

Figure S26. ^{1}H NMR of PCU-1 (400 MHz DMSO-d_6).

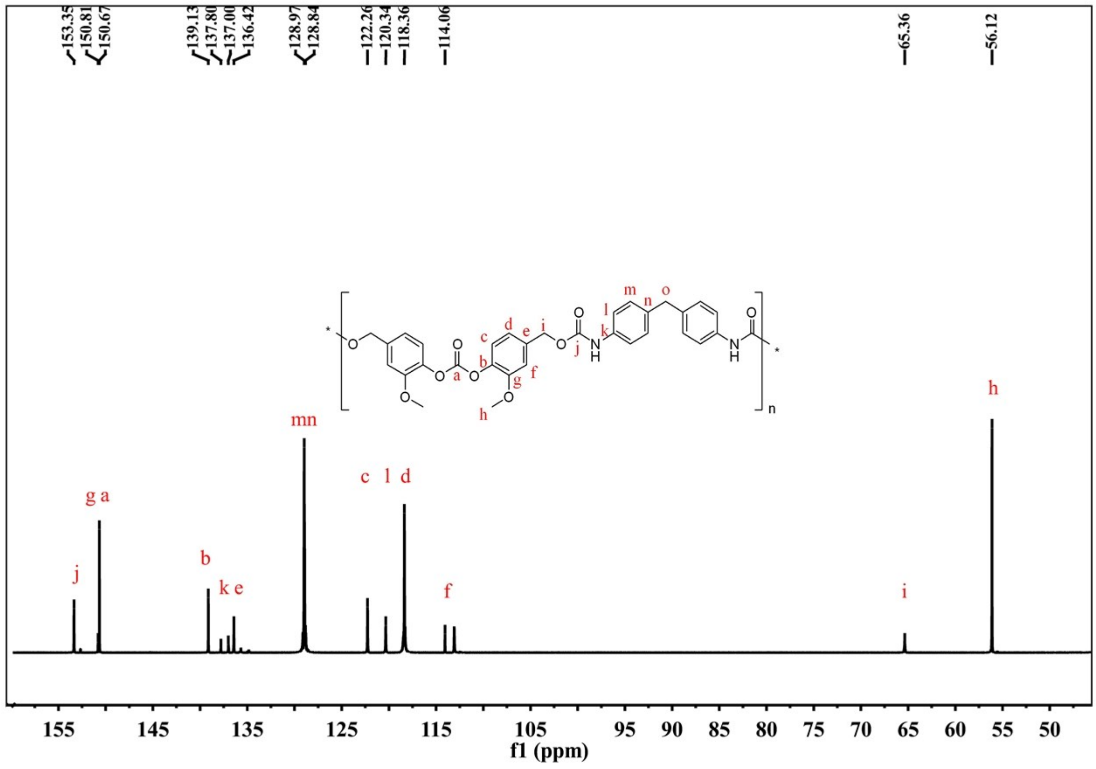

Figure S27. ^{13}C NMR of PCU-1 (101 MHz DMSO-d_6).

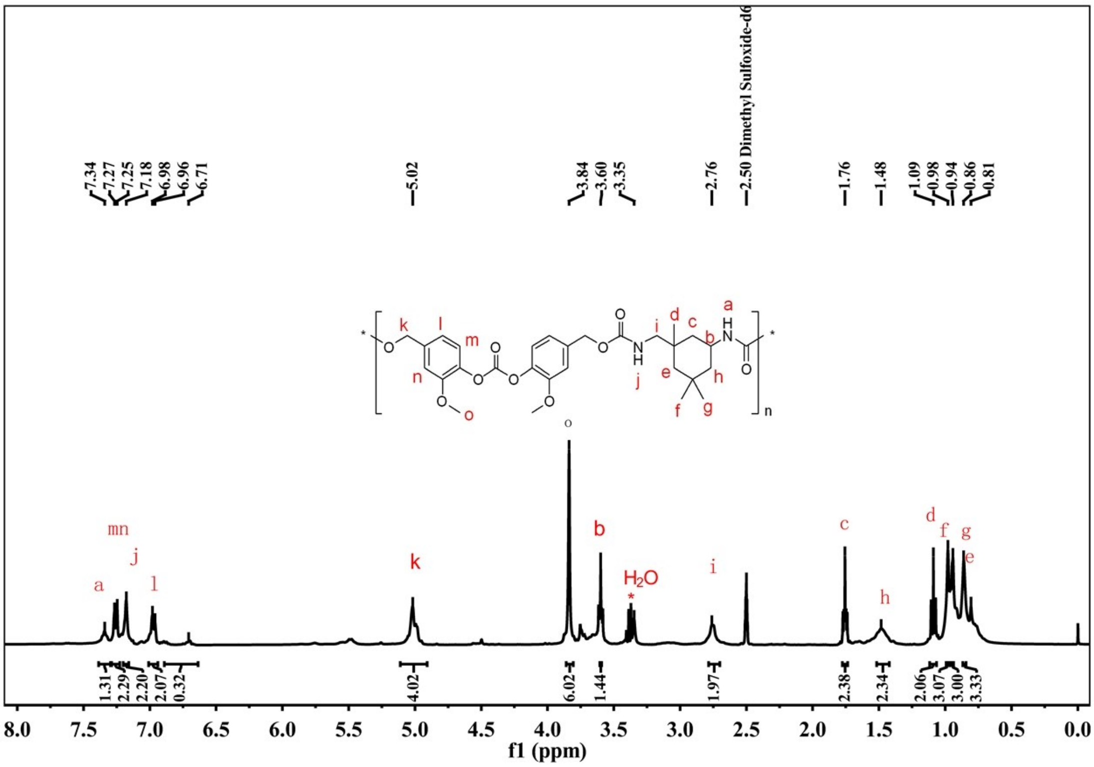

Figure S28. ^{1}H NMR of PCU-2 (400 MHz DMSO-d6).

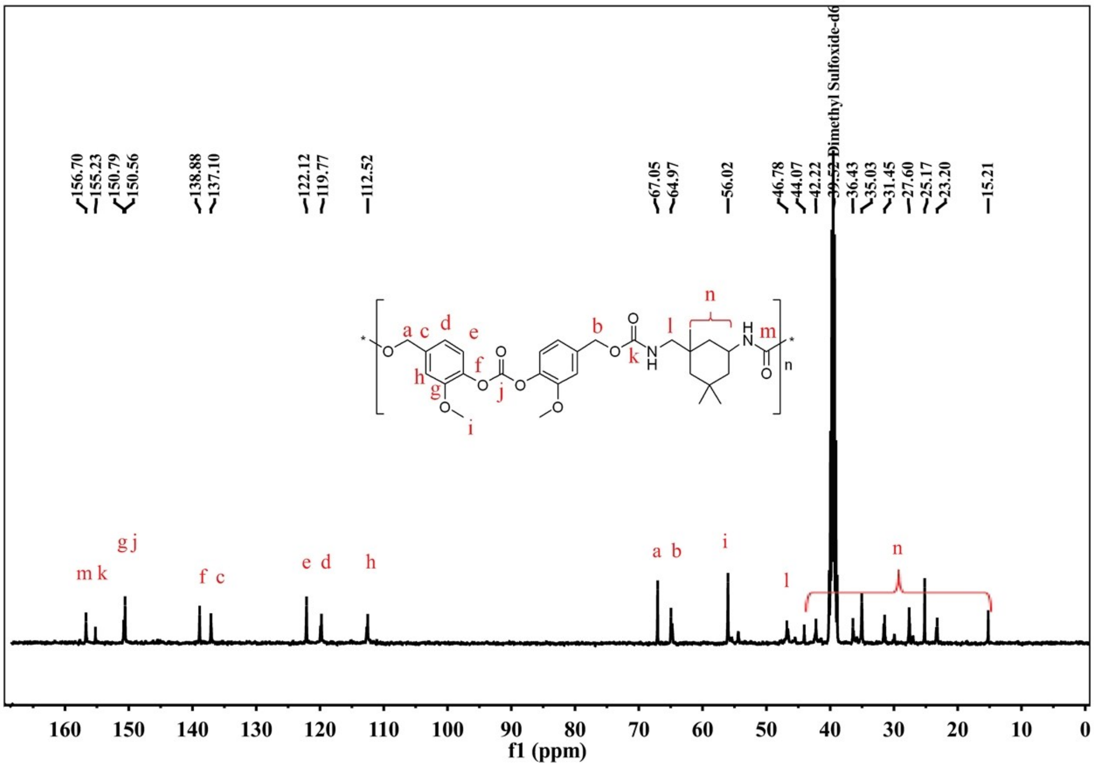

Figure S29. ^{13}C NMR of PCU-2 (101 MHz DMSO-d_6).

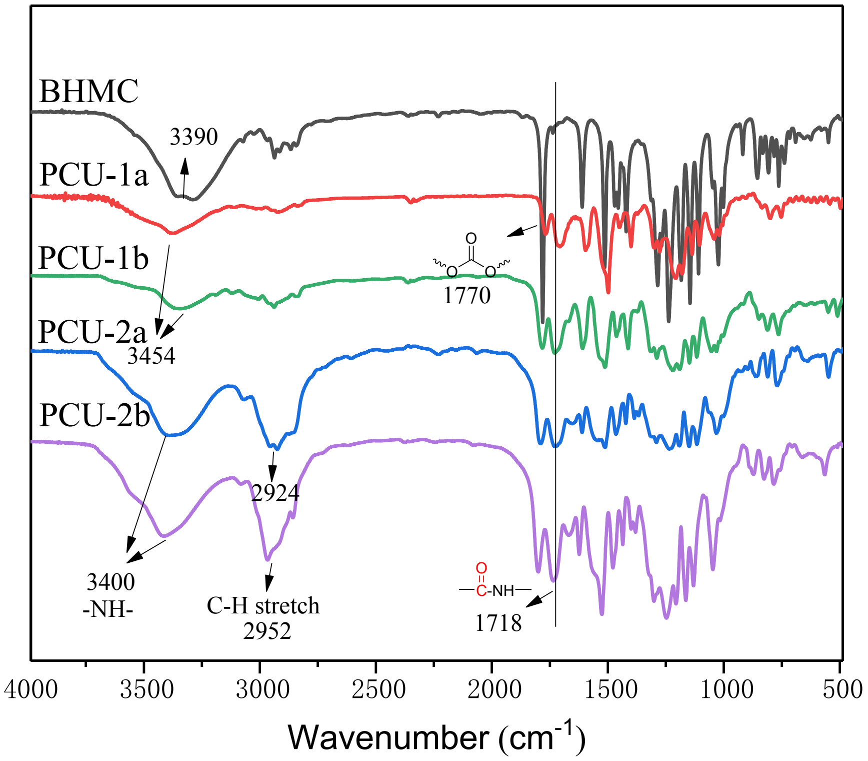

Figure S30. FTIR spectra of BHMC and representative resulting PCUs.

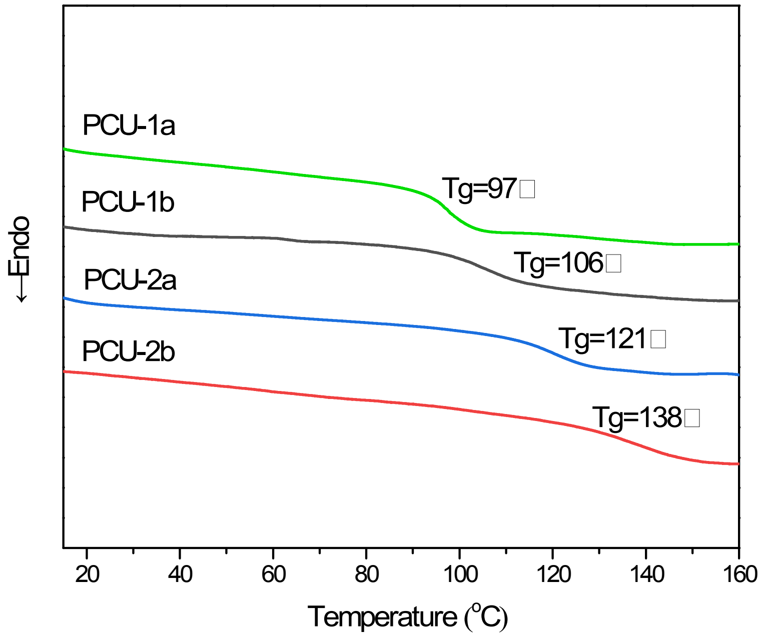

Figure S31. DSC curves of PCUs.

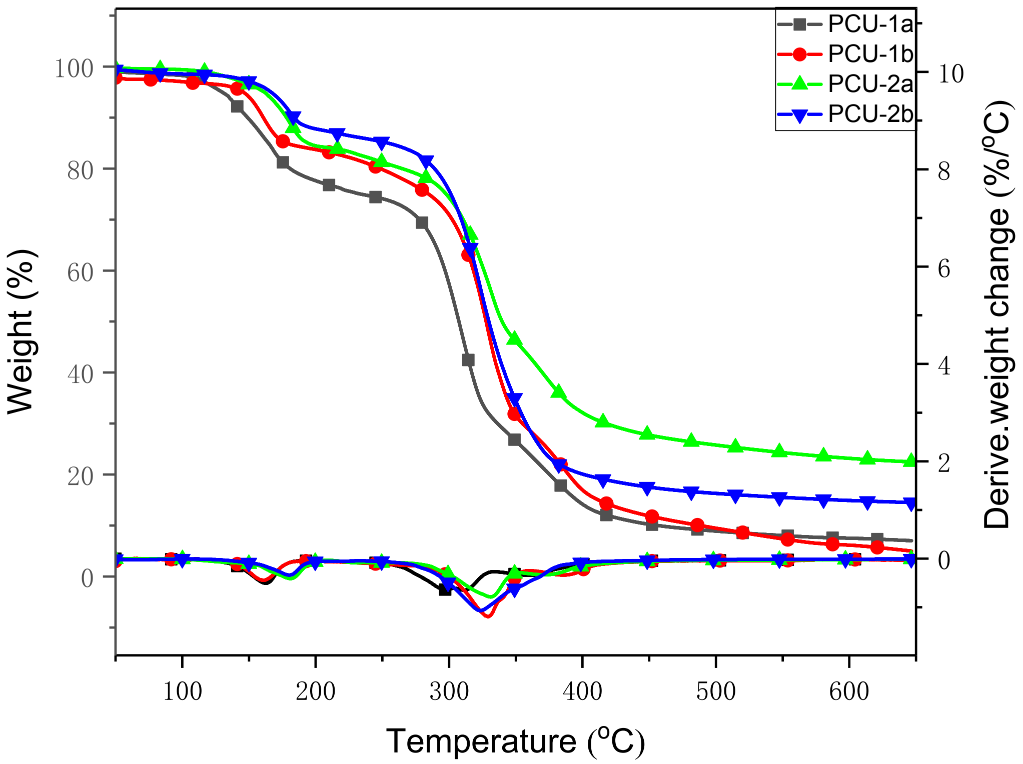

Figure S32. TGA and DTG curves of PCUs.

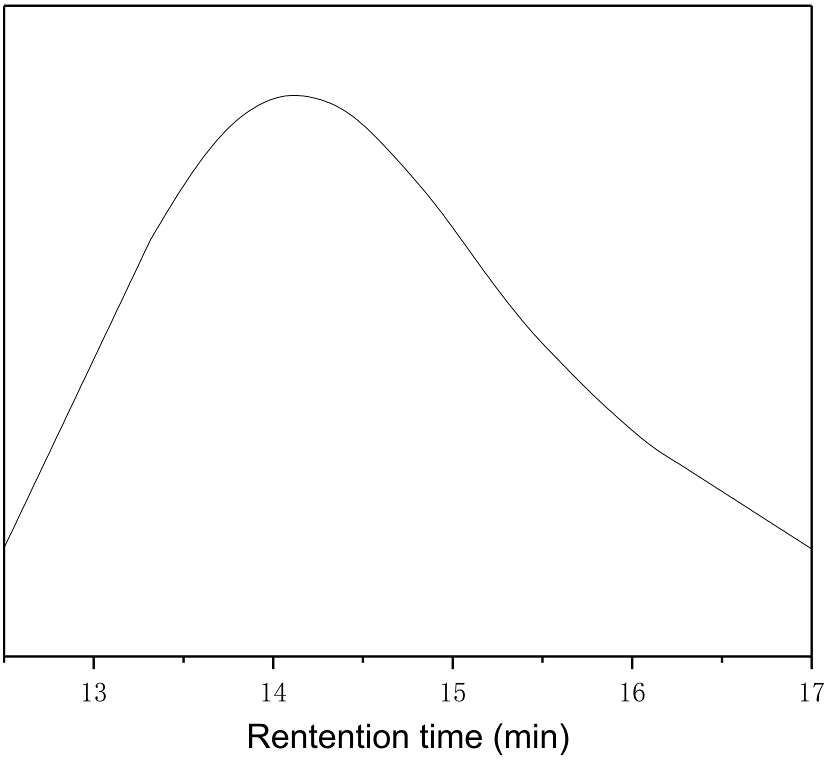

Figure S33. GPC analysis of PCU-1a, M_n=15958 g/mol, PDI=1.84.

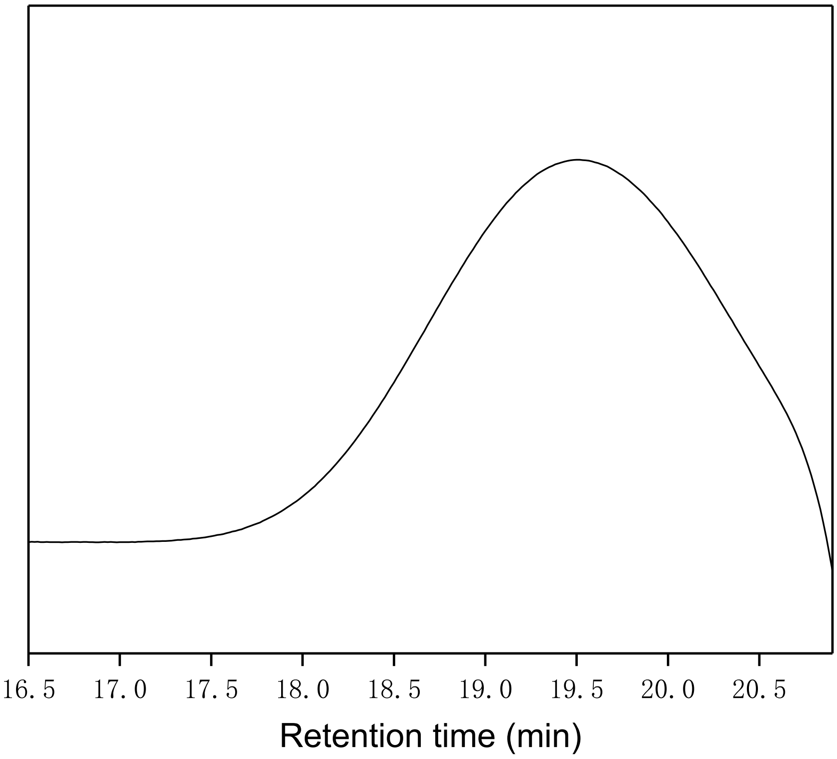

Figure S34. GPC analysis of PCU-1b, M_n=16422 g/mol, PDI=1.36.

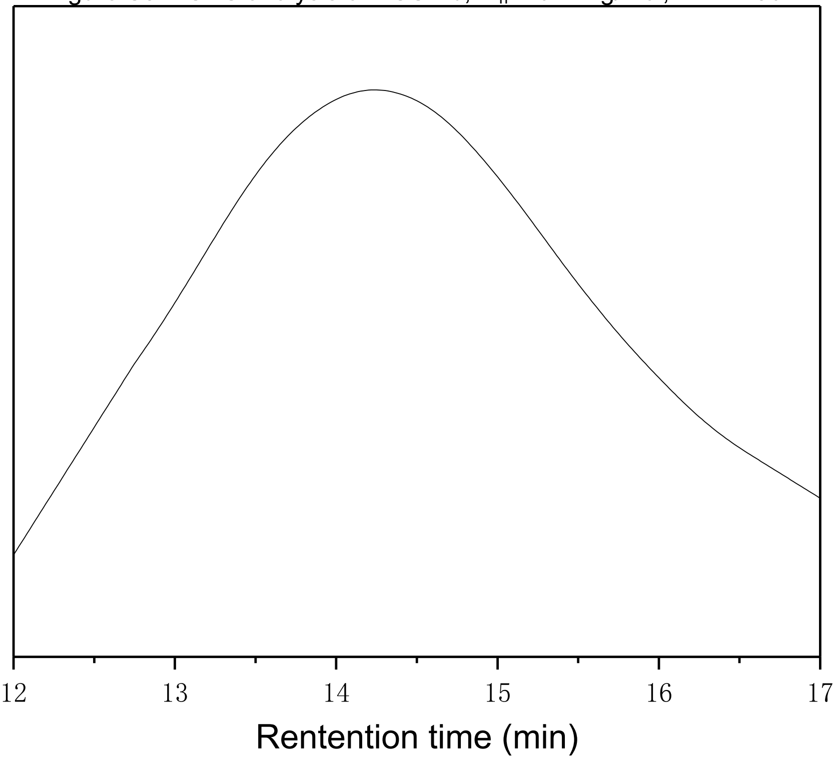

Figure S35. GPC analysis of PCU-2a, M_n=19844 g/mol, PDI=2.17.

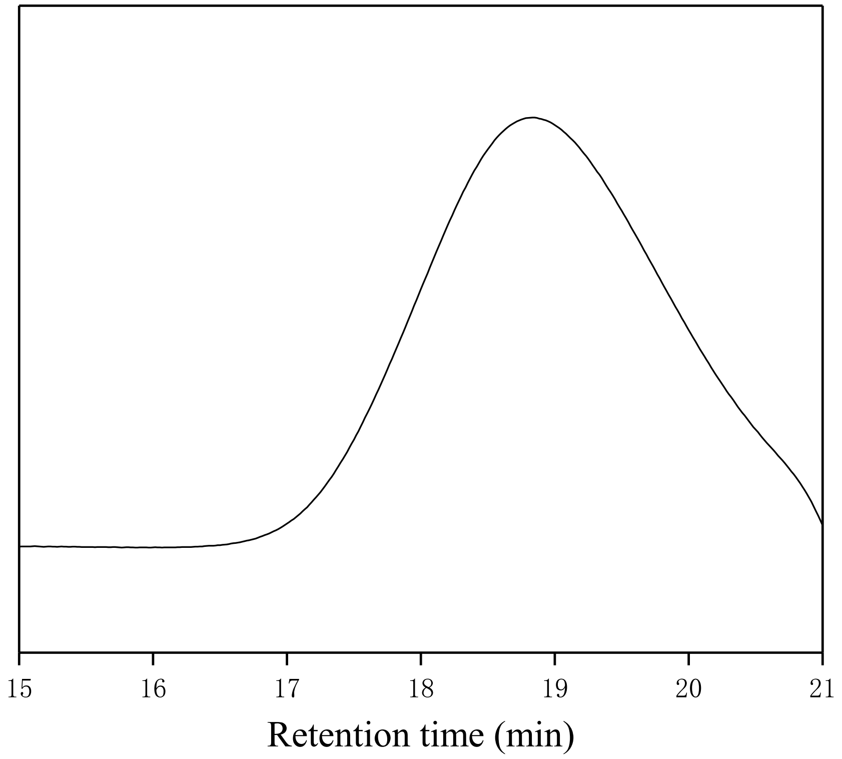

Figure S36. GPC analysis of PCU-2b, M_n=24349 g/mol, PDI=1.61.

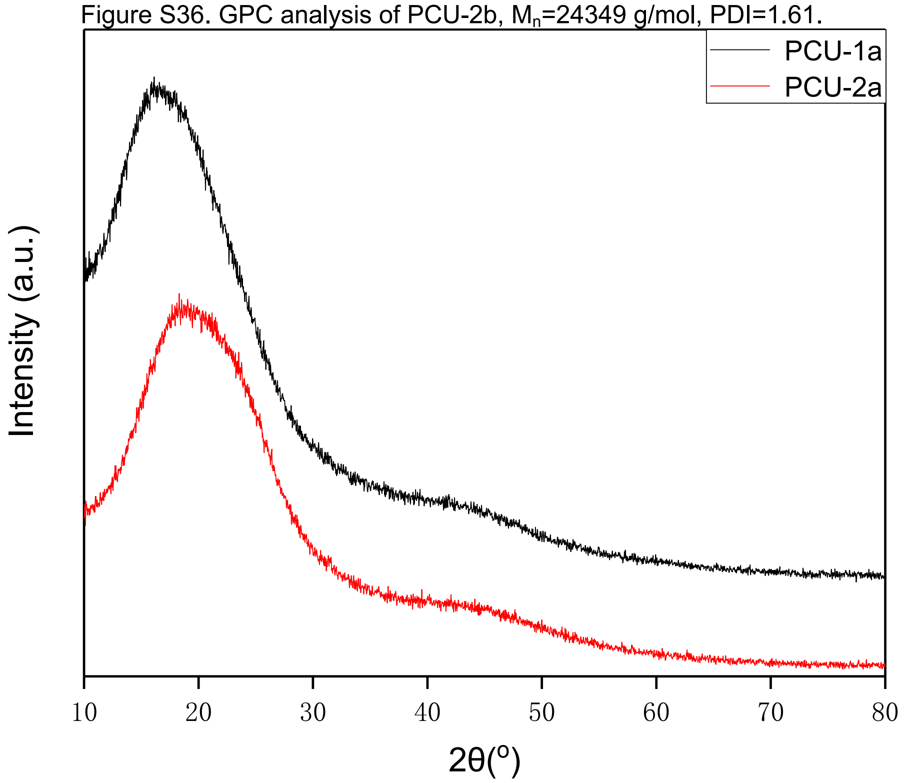

Figure S37. XRD curves of PCUs.

E-factor analysis of the process toward bio-based polyesters

R. A. Sheldon has proposed the environment impact factor (*E*-factor) to quantify the sustainability of a process[1-3]. The *E*-factor is calculated by kilograms of waste generated including 10% of solvent losses divided by kilograms of desired product (Equation 1).

$$\text{E-factor} = \frac{\sum m\ (rawmaterials) + \sum m\ (reagents) + \sum m\ (solvents) \times 10\% - m\ (product)}{m\ (product)}$$

Herein we provide details of our *E*-factor anaylsis of the bio-based polyesters (Tables S1-S6). The overall process *E*-factor of vanillin-based polymer **PCEA-2b**, **PCE-1b** and **PCU-1b** were determinded as 4.350, 12.135 and 8.976, kg/kg, respectively, which are in accordance with Sheldon's analysis of bulk and fine chemicals that have an *E*-factor of 4-50[1-3].

Table S1. Material input-output table for the process toward **PCEA-2b**.

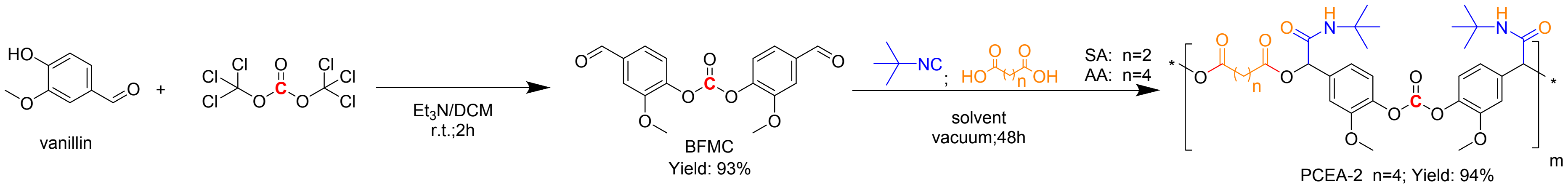

Step number	Input material	Input type	output material	MW (g/mol)	Density (g/mL)	Equiv.	Input mol	input weight (kg)	Input volume (L)	Output weight (kg)	Output mol	Yield
1	vanillin	Raw material		152.15		1	1.673	0.255				
	triphosgene	Raw material		296.75		0.167	0.279	0.083				
	triethylamine	Reagent		101.19	0.728	1.5	2.510	0.254				
	dichloromethane	Solvnet		84.93	1.325			39.750	30.000			
			BFMC	330.29						0.514	1.556	93%
2	BFMC	Intermediate		330.29		1	1.556	0.514				
	Hexanedioic acid	Raw material		146.14		1	1.556	0.227				
	tert-butyl isonitrile	Raw material		83.13	0.735	2	3.112	0.259				
	dichloromethane	Solvnet		84.93	1.325			2.981	2.250			
			PCEA-2b	642.69						1.000	1.556	94.80%

Table S2. E-factor analysis of the process toward **PCEA-2b**.

step number	Raw material (kg)	Reagent (kg)	Solvent	Product (kg)	step E-Factor	E-Factor
1	0.337	0.254	39.750	0.514	7.885	4.052
2	1.000	0.00	2.981	1.000	0.298	0.298
Total	0.823	0.254	42.731	1.000		4.350

Table S3. Material input-output table for the process toward **PCE-1a**.

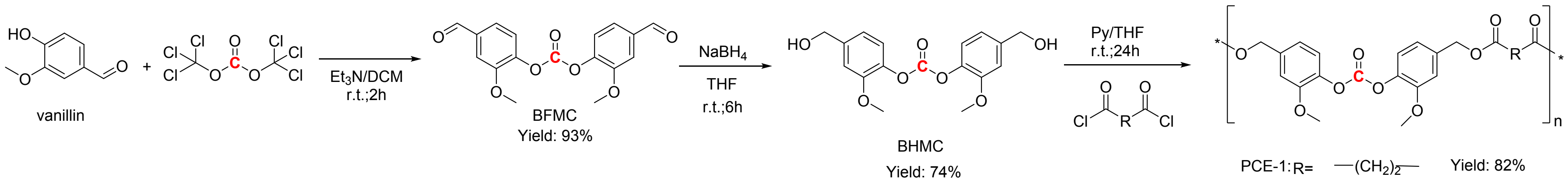

Step number	Input material	Input type	output material	MW (g/mol)	Density (g/mL)	Equiv.	Input mol	input weight (kg)	Input volume (L)	Output weight (kg)	Output mol	Yield
	vanillin	Raw material		152.15		1.000	4.091	0.622				
	triphosgene	Raw material		296.75		0.167	0.682	0.202				
1	triethylamine	Reagent		101.19	0.728	1.500	6.137	0.621				
	dichloromethane	Solvnet		84.93	1.325			92.750	70.000			
			BFMC	330.29						1.257	3.805	93%
	BFMC	Intermediate		330.29		1.000	3.805	1.257				
2	Sodium borohydride	Raw material		37.83		3.000	11.414	0.432				
	tetrahydrofuran	Solvnet		72.11	0.735			7.350	10.000			
			BHMC	334.32						1.094	3.272	86%
	BHMC	Intermediate		334.32		1.000	3.272	1.094				
	Succinyl chloride	Raw material		154.98	1.407	1.000	3.272	0.507				
3	pyridine	Reagent		79.1	0.983	0.022	0.072	0.006				
	tetrahydrofuran	Solvnet		72.11	0.735			7.350	10.000			
			PCE-1a	416.38						1.000	2.402	73.40%

Table S4. E-factor analysis of the process toward **PCE-1a**.

step number	Raw material (kg)	Reagent (kg)	Solvent	Product (kg)	step E-Factor	E-Factor
1	0.825	0.621	92.750	1.257	7.531	9.464
2	1.688	0.000	7.350	1.094	1.215	1.330
3	1.601	0.006	7.350	1.000	1.342	1.342
Total	1.764	0.627	107.45	1.000		12.135

Table S5. Material input-output table for the process toward **PCU-1b**.

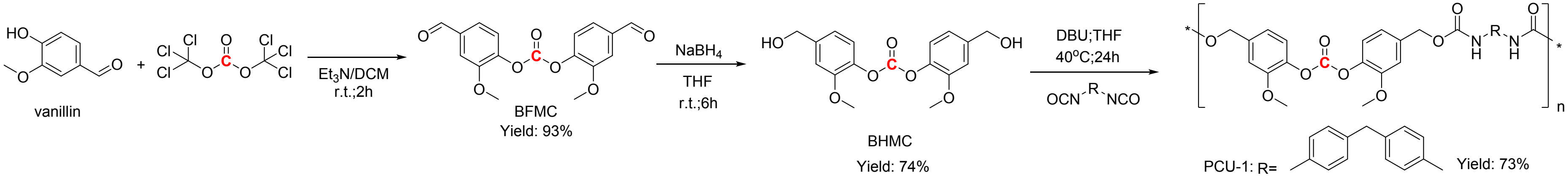

Step number	Input material	Input type	output material	MW (g/mol)	Density (g/mL)	Equiv.	Input mol	input weight (kg)	Input volume (L)	Output weight (kg)	Output mol	Yield
1	vanillin	Raw material		152.15		1.000	3.060	0.466				
	triphosgene	Raw material		296.75		0.167	0.510	0.151				
	triethylamine	Reagent		101.19	0.728	1.500	4.591	0.465				
	dichloromethane	Solvnet		84.93	1.325			66.250	50.000			
			BFMC	330.29						0.940	2.846	93%
2	BFMC	Intermediate		330.29		1.000	2.846	0.940				
	Sodium borohydride	Raw material		37.83		3.000	8.538	0.323				
	tetrahydrofuran	Solvnet		72.11	0.735			5.880	8.000			
			BHMC	334.32						0.818	2.448	86%
3	BHMC	Intermediate		334.32		1.000	2.448	0.818				
	diphenylmethane diisocyanate	Raw material		250.25		1.000	2.448	0.613				
	DBU	Reagent		152.24	1.019	0.030	0.073	0.011				
	tetrahydrofuran	Solvnet		72.11	0.735			7.350	10.000			
			PCU-1b	556.61						1.000	1.797	73.40 %

Table S6. E-factor analysis of the process toward **PCU-1b**.

step number	Raw material (kg)	Reagent (kg)	Solvent	Product (kg)	step E-Factor	E-Factor

1	0.617	0.465	66.250	0.940	7.198	6.766
2	1.263	0.000	5.880	0.818	1.262	1.033
3	1.431	0.011	7.350	1.000	1.177	1.177
Total	1.553	0.476	79.48	1.000		8.976
